# Circulating MicroRNAs Associated with Changes in the Placenta and Their Possible Role in the Fetus During Gestational Diabetes Mellitus: A Review

**DOI:** 10.3390/metabo15060367

**Published:** 2025-06-03

**Authors:** Ninna Leslie Trejo-Gonzalez, Martin Palomar-Morales, Luis Arturo Baiza-Gutman, Guadalupe Diaz-Rosas, Clara Ortega-Camarillo, Alejandra Contreras-Ramos

**Affiliations:** 1 Postgraduate in Biological Sciences, National Autonomous University of Mexico, Mexico City C.P. 04510, Mexico; ninnatrejo1988@gmail.com; 2 Molecular Biology Research Laboratory, Children’s Hospital of Mexico Federico Gomez (HIMFG), Mexico City C.P. 06720, Mexico; gudiro2@yahoo.com.mx; 3 Department of Biology, Faculty of Higher Studies Iztacala, National Autonomous University of Mexico, Mexico City C.P. 54090, Mexico; palomarmoralesmartin@gmail.com; 4 Laboratory of Developmental Biology, Morphology and Function Unit, Faculty of Higher Studies Iztacala, National Autonomous University of Mexico, Mexico City C.P. 54090, Mexico; labaiza@unam.mx; 5 Medical Research Unit in Biochemistry, Specialties Hospital, National Medical Center SXXI, Instituto Mexicano del Seguro Social, Mexico City C.P. 06720, Mexico

**Keywords:** gestational diabetes, maternal circulation, miRNAs, placenta, amniotic fluid, umbilical cord blood, embryonic alterations

## Abstract

MicroRNAs (miRs) are epigenetic regulators of several metabolic diseases, including gestational diabetes mellitus (GDM). Objectives: Following a systematic review, we propose a pattern of key circulating miRs associated with placental changes and their potential role in the fetus. Methods: A systematic investigation of studies published between January 2011 and July 2024 was conducted in the PubMed, ScienceDirect, Trip Database, and Wiley databases. A total of 90 articles were analyzed. Results: Two hundred twenty-six circulating microRNAs were identified in women with GDM, and fifty miRs were validated by PCR, with miRs-16-5p, -29a-5p, and -195-5p being the most frequently reported. Interestingly, miR-16-5p was also expressed in the placenta but not in umbilical cord blood or amniotic fluid. Conversely, miR-126-3p was expressed in circulation, the placenta, umbilical cord blood, and amniotic fluid. Several reports describe high expression levels of miR-518d in maternal circulation, umbilical cord blood, and placenta. Controversial results regarding the expression of miR-29a-3p, -137, and -148a-3p were identified when comparing umbilical cord blood and the placenta. Conclusions: In silico analyses suggest that the miR-29 family, as well as miRs-16-5p, -126-3p, -195-5p, and -518b, may be involved in alterations in the heart, brain, and kidneys in the embryo when exposed to a hyperglycemic environment.

## 1. Introduction

Gestational diabetes mellitus (GDM) is a global public health issue. According to the International Diabetes Federation (IDF), in 2021, 21 million children (1 in every 6 newborns worldwide) were born from pregnancies complicated by GDM, with hyperglycemia occurring at some point during their intrauterine development [1].

GDM is typically diagnosed for the first time during the second or early third trimester of pregnancy using a glucose tolerance test. Diagnostic thresholds are a fasting glucose concentration greater than 92 mg/dL, 180 mg/dL at 1 h, or 153 mg/dL at 2 h after a glucose challenge [2]. Insulin resistance, driven by the action of antagonistic hormones such as placental lactogen, cortisol, progesterone, prolactin, estrogen, and growth hormone, leads to pancreatic β-cell dysfunction and the onset of GDM [3]. GDM triggers various complications in the fetus and newborn, including congenital malformations, preterm birth, and macrosomia. Approximately 4% of fetuses experience congenital anomalies that may result in death or increase the risk of intrauterine complications and spontaneous abortions [1]. Furthermore, children born to diabetic mothers are at an elevated risk of developing type 2 diabetes (T2D), obesity, dyslipidemia, and other related conditions [3,4].

MicroRNAs, on the other hand, are noncoding RNAs that regulate gene expression at the post-transcriptional level. These small molecules are considered biomarkers for specific tissues or diseases due to their stability, which allows for quantification and identification in multiple tissues such as blood, serum, and plasma, and in non-invasive samples such as urine, from various pathological conditions [5].

In 2010, Zampetaki et al. pioneered the identification of an expression profile of circulating miRNAs in T2D [6]. Since then, efforts to identify molecular signatures associated with hyperglycemia-induced pathologies have persisted. Numerous studies have documented the expression profiles of genes in blood [7], plasma [8,9], amniotic fluid [10], and placenta [11,12,13] from patients with GDM. In these studies, dysregulation of miR-330-3p [7], miR-16-5p, miR-17-5p, miR-20a-5p [14], and miR-138 [11] has been highlighted. Some studies have even proposed the association of the expression of some miRNAs with the health status of women during GDM [15,16,17,18] or the newborn [19].

However, the abundance of information demands further analysis to allow for a better interpretation of the importance of changes in miRNA expression in the mother and in the baby, derived from GDM, which would allow for a molecular diagnostic and/or prognostic signature to be established.

In order to address this gap, a systematic review was conducted to analyze and integrate international literature from the last fourteen years on miRNAs associated with the health status of mothers with GDM and their offspring.

## 2. Materials and Methods

Study Protocol: A bibliographic search was conducted in the PubMed, ScienceDirect, Trip Database, and Wiley databases. The Boolean terms “microRNAs” OR “microRNA” OR “miRNA” OR “miRNAs” OR “miR” AND “gestational diabetes” OR “gestational diabetes mellitus” OR “diabetic pregnancy” were used. Before the main search for articles that would form the core material of this work, an exploratory search was performed, covering the years 1994 to 31 July 2024. This timeframe was chosen because it includes the discovery of miRNAs, with the earliest study meeting our inclusion criteria published in 2011. The primary research focused on studies published from January 2011 until July 2024. For research in ScienceDirect, the filter “original articles” was applied; for Wiley, the “journals” filter was used.

Inclusion Criteria: Original articles utilizing whole blood, plasma, serum, placenta, umbilical cord blood, or amniotic fluid from women diagnosed with GDM, with the main objective of determining miRNA expression profiles or analyzing specific miRNA functions.

Exclusion Criteria: Studies involving in vivo animal models or those focusing on pregestational diabetes mellitus, preeclampsia, or preterm births.

Data Collection: A standardized form was developed to collect data, including the year, author, title, journal, DOI, study objective, and characteristics of the study population. In the results section, data on expression profiles, functional studies, organs or cell lines used, and implicated signaling pathways were grouped.

Results Analysis: To identify patterns, similarities, or concordances, tables and graphs were created based on the analyzed studies, providing better visualization. miRNAs with the highest number of reports were used to search for target genes using QIAGEN IPA (QIAGEN Inc., https://digitalinsights.qiagen.com/IPA (Aarhus C and Denmark) (accessed on 02 February 2025)), focusing on genes associated with cardiac, cerebral, and limb anomalies in the fetus. This information was presented as a signaling pathway.

## 3. Results

### 3.1. Literature

**Figure 1:**
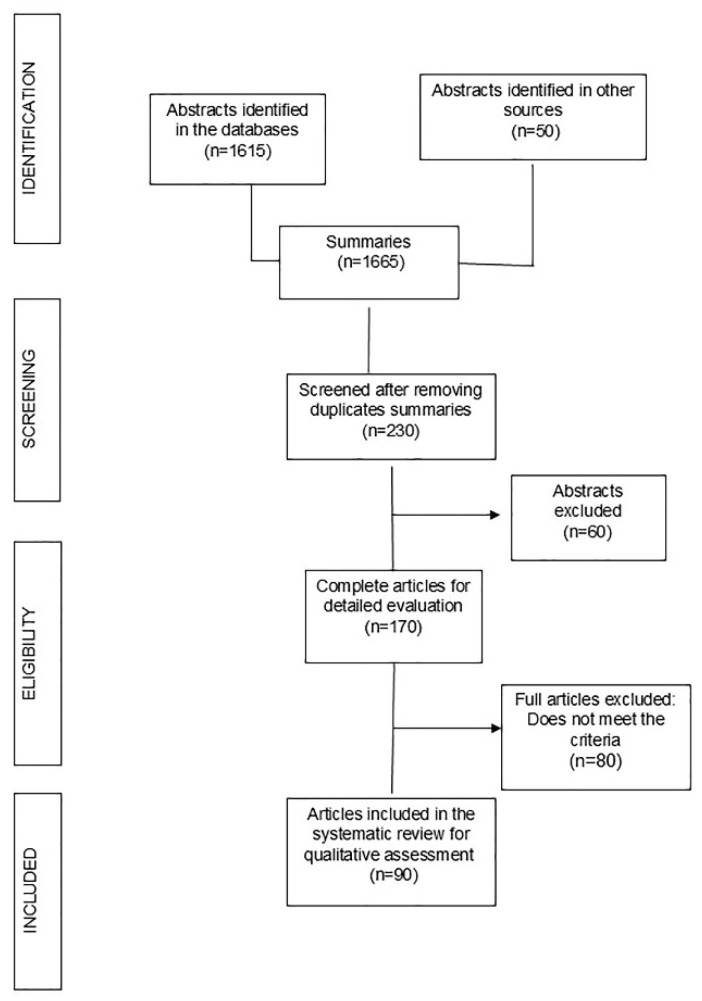
PRISMA flowchart for item selection.

Figure 1 shows the search process across the different databases. After eliminating duplicate articles and reviewing abstracts, ninety articles were included and analyzed in this review [5,7,8,9,10,11,12,13,14,15,16,17,18,19,20,21,22,23,24,25,26,27,28,29,30,31,32,33,34,35,36,37,38,39,40,41,42,43,44,45,46,47,48,49,50,51,52,53,54,55,56,57,58,59,60,61,62,63,64,65,66,67,68,69,70,71,72,73,74,75,76,77,78,79,80,81,82,83,84,85,86,87,88,89,90,91,92,93,94].

### 3.2. Characteristics of the Studies

The general characteristics of the study populations are presented in Table S1 (Supplementary Tables https://1drv.ms/f/s!AkeZUt9if-achoYiraRx4JIWM73kvw?e=yjs9xW). Of the ninety articles analyzed:

- A total of 70% of the studies examined blood, serum, or plasma.
- A total of 94.59% reported the number of patients included. Among these, a total of 3782 women were diagnosed with GDM, and 3817 served as controls (CTR).
- The minimum number of women with GDM or controls included in the studies was 5, while the maximum was 236 women with GDM and 204 controls.
- A total of 30% of the articles reported participant age, with a minimum range of 21–27 years and a maximum of 35 years.
- A total of 41% of the articles referenced the body mass index (BMI), with values ranging from 21 to 34.
- Data on glucose tolerance tests were included in 40% of the reports. Additionally, some articles described neonatal weight.

### 3.3. Circulating MiRNA Profiles in Women with Gestational Diabetes

Although the expression profiles of all miRNAs were reported in nineteen articles, it was not possible to obtain the full set of miRNAs, as some studies did not provide complete data. Of these, three articles evaluated miRNAs in the first trimester, twelve articles in the second trimester, and one article in the third trimester. Additionally, three articles conducted longitudinal studies, evaluating all three trimesters. Approximately 226 circulating microRNAs were identified at least once in the serum, plasma, or blood of women with GDM. Of these, only fifty miRNAs were validated by PCR (Table S2, (Supplementary Tables https://1drv.ms/f/s!AkeZUt9if-achoYiraRx4JIWM73kvw?e=yjs9xW)).

**Figure 2:**
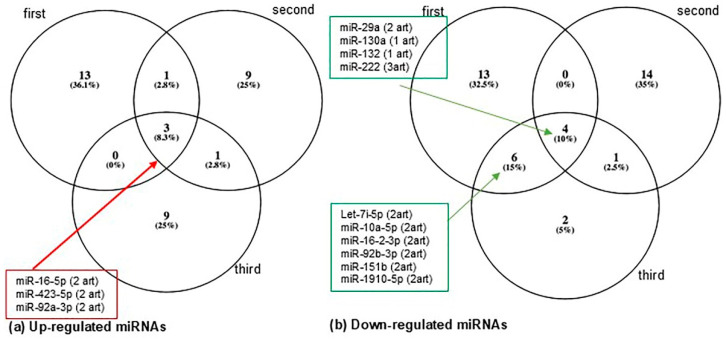
Venn diagram. The most frequently reported circular miRNAs during the first, second, and third trimesters in women with gestational diabetes mellitus are shown. (**a**) Up-regulated miRNAs: miR-16, miR-423-5p, and miR-92a-3p; (**b**) down-regulated miRNAs: miR-130a, miR-132, miR-222, and miR-29a.

Figure 2a groups the miRNAs by trimester. The expression of miR-16-5p, -92a-3p, -195-5p, and -423-5p was identified across all three trimesters by two independent studies. Meanwhile, miR-29a, -130a, -132, and -222-5p were down-regulated across all trimesters (Figure 2b). Additionally, six miRNAs (Let-7i-5p, -10a-5p, -151b, -16-2-3p, -92b-3p, and -1910-5p) were reported to be down-regulated between the first and third trimesters (Figure 2b).

Furthermore, Table S3 summarizes forty-four studies that analyzed miRNAs using real-time qPCR. Among these, the most frequently reported miRNAs were miR-16-5p, -29a-5p, -122-5p, -132-5p, -222-5p, and -195-5p (Supplementary Tables https://1drv.ms/f/s!AkeZUt9if-achoYiraRx4JIWM73kvw?e=yjs9xW).

### 3.4. MiRNAs Identified in Maternal Circulation vs. Placenta

**Figure 3:**
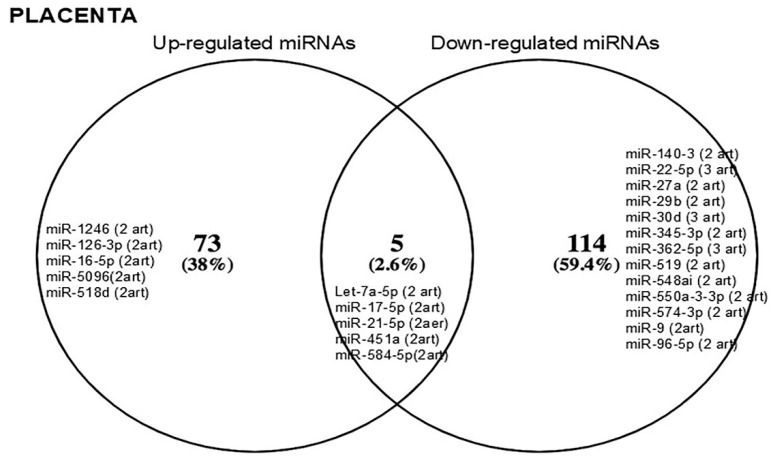
Venn diagram. The most frequently reported miRNAs in the placenta of women with gestational diabetes mellitus are shown. On the left are the up-regulated miRs, and on the right are the down-regulated miRs. At the intersection, miRNAs whose expression is still controversial are shown. The parentheses indicate the number of reports.

**Figure 4:**
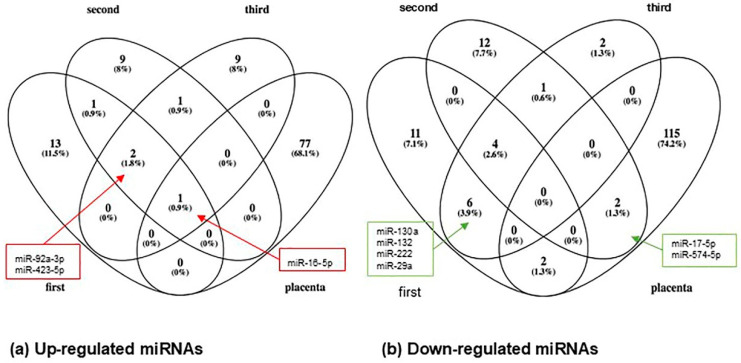
Comparative Venn diagram. Clustering of circulating miRs and placental miRs determined in women with gestational diabetes mellitus. (**a**) In the up-regulated miRNAs, we can highlight miR-16-5p, which was reported in the circulation during the three trimesters and in the placenta. (**b**) In the down-regulated miRNAs, note that miR-17-5p and miR-574-5p were reported in the second trimester and in the placenta.

The evaluation of 192 miRNAs in the placenta of women with GDM using PCR or RNA-seq was conducted in thirty-four studies (Table S4 Supplementary Tables https://1drv.ms/f/s!AkeZUt9if-achoYiraRx4JIWM73kvw?e=yjs9xW). Of these, five miRNAs (Let-7a-5p, -17-5p, -21-5p, -451a, and -584-5p) were described as having differential expression in at least two independent studies (Figure 3). Interestingly, the increased expression of miR-16-5p (reported in two articles) in the placenta was similar to that observed in circulation (Figure 4a). Meanwhile, the down-regulation of miR-17-5p and -574-5p in the placenta was also observed in the second trimester (Figure 4b).

### 3.5. Functional Studies

Functional studies of miRNAs, their potential target genes, and biological implications were conducted through in vitro transfection (Table 1). Most functional studies (27 in total) were performed in endothelial cell lines derived from umbilical cord or placental tissue (e.g., HUVEC, HUVEC-GDM, HRT-8/SVneo/BeWo, villous cytotrophoblasts, and JEG-3). The results demonstrated the expression of genes related to glucose metabolism (GLUT1, SLC2A5, HK2, PPARγ, and PGC1α) as targets of miR-22 and -518d [5,58,89]; intracellular trafficking (RAB8A); antioxidant enzymes such as glutathione reductase (RAB8A); cell migration (TBL1X and HIF3A); viability, proliferation, or apoptosis (PAK1, NKX6-1, and BAK); and insulin signaling or resistance associated with miR-140 (AMPKα2, IR-α, IRS-1, and IGF1R), among others [88,93]. Other authors performed functional studies using pancreatic beta cell lines (MIN6 and INS1) to identify targets of miR-190b and miR-96. NKX6-1 and PAK1 genes were identified as targets of miR-190b and miR-96, respectively [80,86]. Down-regulation of NKX6-1 affects B cell proliferation and insulin secretion. Inhibition of PAK1 inhibits insulin secretion and glucose uptake by decreasing GLUT4 translocation [80]. A few studies utilized human leukemia cell lines transfected with miR-657 and miR-6869-5p, identifying IL-37 and PTPRO as target genes involved in the anti-inflammatory response [77,78]. In the case of miR-345, independent studies indicated its role as a regulator of apoptosis, cell viability, and migration through BAK1. Additionally, two studies demonstrated the involvement of miR-518d in cellular proliferation via the regulation of PPAR-α expression [89,94].

### 3.6. MiRNAs in Umbilical Cord Blood and Amniotic Fluid vs. Placenta and Circulation in Gestational Diabetes

Table 2 presents miRNAs identified in umbilical cord blood and amniotic fluid, compared with their reports in the placenta and maternal circulation in women with GDM. In this context, increased expression of miR-126-3p was identified in umbilical cord blood, amniotic fluid, the placenta, and maternal circulation. Additionally, the expression of miR-518d was reported in umbilical cord blood, the placenta, and circulation. For miR-96-5p and miR-345-3p, deregulation observed in umbilical cord blood was also reported in the placenta. Controversial results were described for miR-29a-3p, -137, and -148a-3p when comparing their expression in umbilical cord blood vs. the placenta or circulation. In the case of amniotic fluid, the expression of miR-138, -197-3p, and -199a-3p was also reported in the placenta. Furthermore, the expression of miR-185-5p in amniotic fluid was also observed in maternal circulation (Table 3).

### 3.7. Identification of Candidate MiRNAs and Their Target Genes (miR-16-5p, -126-3p, -185-5p, -195-5p, -222-3p, -518b, and the miR-29 Family) Related to Neonatal Development

**Figure 5:**
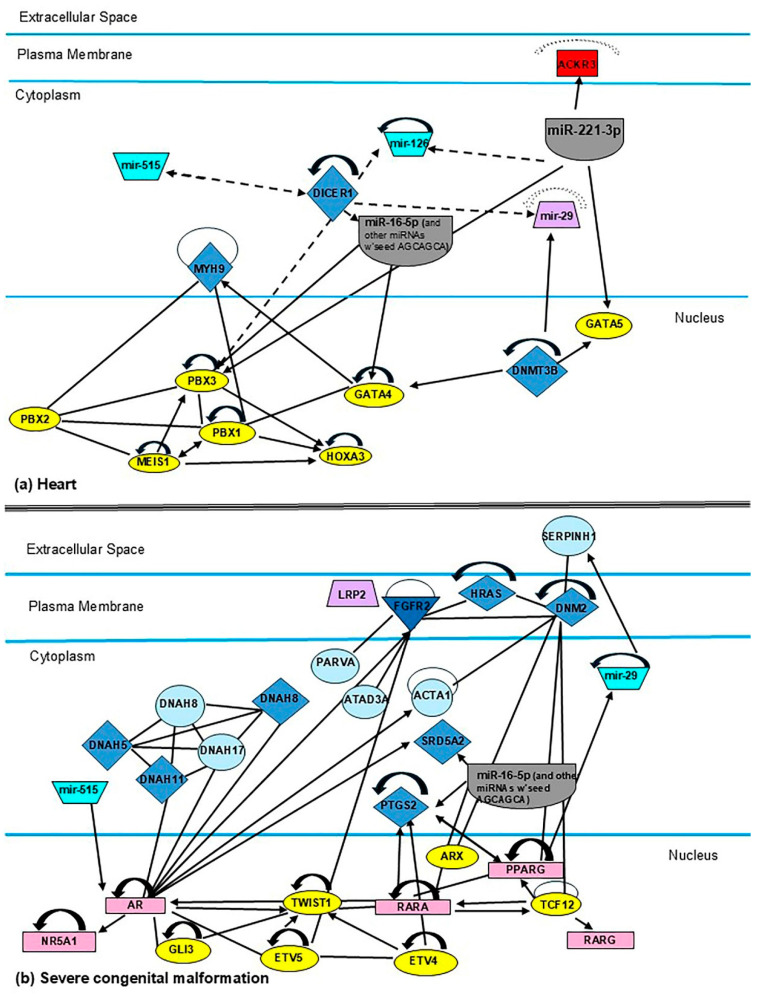

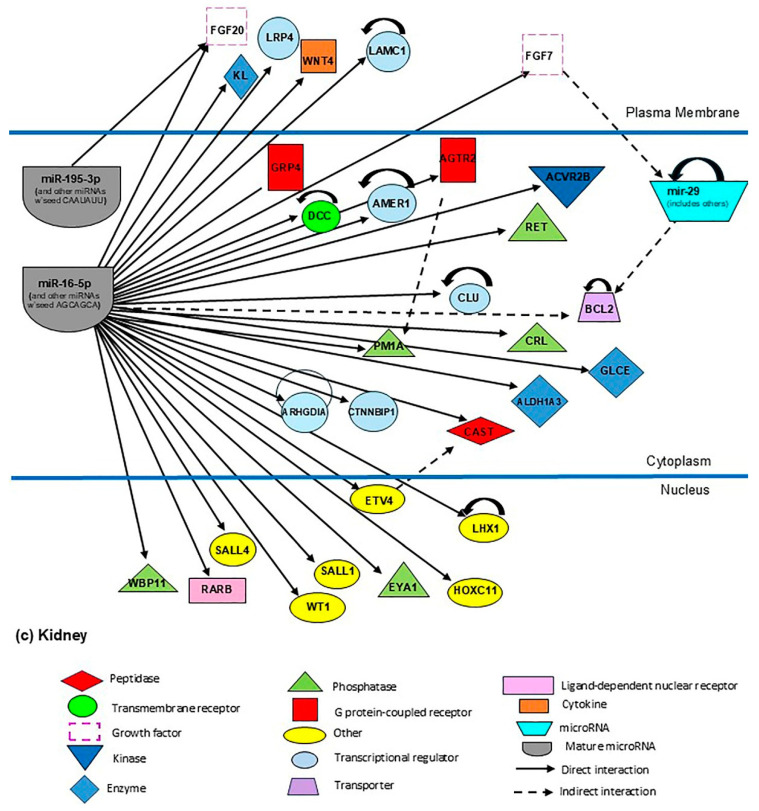
Schematic of candidate miRNA regulation. Targets and functions of miR-16, miR-29, miR-126, and miR-515 in development in the heart (**a**), severe congenital malformation (**b**), and the kidneys (**c**).

To determine the significance of miRNAs frequently reported in the scientific literature in relation to fetal development, the The networks were generated through the use of QIAGEN IPA (QIAGEN Inc., https://digitalinsights.qiagen.com/IPA). Gene networks were established to intuitively analyze the relationship of miR-16-5p, -126-3p, -185-5p, -195-5p, -222-3p, -518b, and the miR-29 family, which are implicated in the development of the heart, congenital malformations, and kidneys, as shown in Figure 5a–c, respectively. These analyses highlight the biological roles of miR-16-5p, -126-3p, -195-5p, -518b, and the miR-29 family in neonatal development.

## 4. Discussion

Numerous studies have described miRNA profiles in women with GDM; however, only a few have focused on the relationship between these miRNAs and alterations in neonatal development. Consequently, controversies persist in the findings. These discrepancies may be linked to study methodologies and/or analysis criteria. In this review, we focused on miRNAs expressed in maternal circulation and the placenta and compared them with those identified in umbilical cord blood and amniotic fluid. Concordances were found in reports on the expression of miR-16-5p, miR-29b-3p, and miR-222-3p in the plasma of women with GDM. Notably, miR-16-5p was also described in the placenta of women with GDM. Furthermore, miR-126, miR-195-5p, miR-29a-3p, and miR-518d, reported in serum and the placenta, were also identified in umbilical cord blood. However, in silico analyses suggest that miR-16-5p, -126-3p, -195-5p, -518d, and the miR-29 family have significant biological implications for offspring development. We consider that the miRNAs proposed in this study could be targets for future studies that address whether changes in their regulation could affect the final fate of the mother–child binomial affected by GDM.

### 4.1. Scope of Case Records

Studies on gestational diabetes have sparked worldwide interest due to the potential complications it poses for both mothers and children in the future. The prevalence of gestational diabetes varies according to the population studied, and ethnicity plays an important role in the genetic predisposition to the disease [95]. It is worth mentioning that the most recent IDF report [1] indicates that the Western Pacific region has the highest number of people diagnosed with type 2 diabetes, while the region with the worst prognosis for 2045 is South Africa, where a 134% increase in the prevalence of the disease is predicted. In addition, the regions of Central and Northern Africa are predicted to experience an 87% increase. However, in this review, studies conducted in China were highlighted, but the lack of information on the study population and other countries did not allow for any miR associated with the population or region to be determined.

It is worth noting that the analyzed articles do not report all risk factors for GDM, which are crucial for determining whether a mother will develop diabetes and its consequences for both mother and child. Maternal age is a significant risk factor, as the likelihood of glucose intolerance and GDM increases with age [96]. Systematic evaluations of maternal weight, waist circumference, height, and even placental weight can help determine the health status of both mothers and fetuses. For instance, maternal size affects a newborn’s body mass index (BMI) [49]. Maternal obesity has been associated with a higher likelihood of preterm birth and macrosomia, as well as an increased risk of the child developing obesity and metabolic syndrome [31,72]. Thus, it would be important to include all clinical parameters to perform association or correlation analyses with miRNA expression profiles. This approach would support the utility of miRNAs as biomarkers for early and late diagnosis and risk prediction in women with GDM, as well as the potential consequences for the neonate. These examples demonstrate that some parameters may be relevant in determining the likelihood of developing GDM. However, the articles analyzed in this review failed to establish an association between health status and microRNAs.

### 4.2. Biological Implications of the MiR-29 Family, MiR-16-5p, -126-3p, -195-5p, and -518b in the Mother–Child Binomial Affected by Gestational Diabetes Mellitus

#### 4.2.1. MiR-16-5p

MiR-16-5p, along with others, is part of the circulating miRNA profile identified in the serum and plasma of women with GDM [14,35,48,55]. Notably, miR-16 levels were detected as early as the first trimester and significantly increased in the plasma of women with GDM [8,75]. An increased expression of miR-16-5p during GDM results in aberrant insulin function, leading not only to impaired glucose metabolism in the mother but also to an imbalance in growth factors critical for fetal development [38]. The combined expression of miR-16-5p with miR-20a-5p, -145-5p, -146a-5p, -181a-5p, -342-3p, and -574-3p allowed for the identification of 42.68% of pregnancies in which neonates presented fetal growth restriction [15]. In placental tissue, independent studies by Marei et al. [19] and Zhang et al. [97] revealed that miR-16-5p expression correlates directly with fetal macrosomia and increased birth weight. However, experimental studies specifically examining miR-16-5p in GDM remain limited. Other studies have experimentally demonstrated that miR-16-5p regulates the cell cycle and induces apoptosis [75]. It affects the insulin/PI3K-Akt pathway in hepatic tissue, with significant consequences for glucose and lipid metabolism [98]. Furthermore, it modulates angiogenesis by regulating VEGF and its receptor expression [99]. Interestingly, while miR-16-5p has not been identified in umbilical cord blood or amniotic fluid, in silico analyses suggest that miR-16-5p may be involved in various embryonic alterations under hyperglycemic conditions. For example, in the heart, miR-16-5p regulates the mRNA expression of PBX3 and GATA4, whose deficiency has been linked to outflow tract defects and impaired cardiac function, respectively. In the brain, miR-16-5p inhibits MEOX1, which plays a role in somatogenesis and is specifically involved in sclerotome formation. It also regulates the transcription factor ETV4, which is implicated in sensory neuron innervation. In the kidneys, miR-16-5p appears central to renal development, regulating approximately 31 target genes, including fibroblast growth factor 7 (FGF7), which in turn affects miR-29 expression. Therefore, miR-16-5p may be one of the most significant microRNAs indirectly influencing fetal development (Figure 5).

#### 4.2.2. MiR-126-3p

MiR-126-3p was expressed both in maternal circulation and in the placenta of women with GDM [77,100]. Interestingly, this miRNA was also reported in umbilical cord blood and amniotic fluid during GDM [62]. Experimental data suggest that miR-126-3p regulates angiogenesis. For instance, in a model of fetal growth restriction induced by a low-protein diet, increased miR-126-3p expression and decreased VEGF expression were observed in the lungs, affecting angiogenesis and fetal lung development [101]. Other studies revealed that miR-126-3p promotes angiogenesis, stimulates granulosa cell proliferation, and reduces apoptosis by inhibiting the PI3K/Akt/mTOR pathway [102]. This contributed to the recovery of early ovarian failure induced by cisplatin [103]. Despite these findings, no functional studies have demonstrated the central role of miR-126-3p in fetal development during GDM. Additionally, in silico studies did not demonstrate an association between miR-126-3p and the development of the heart, pancreas, kidneys, brain, or limbs in the fetus during GDM (Figure 5).

#### 4.2.3. MiR-195

The increase in miR-195 levels has been associated with altered fasting glucose levels as well as glucose levels at 1 and 2 h post-glucose load in women with GDM. Consequently, it has been proposed as a prognostic biomarker by Wang et al. [76]. This miRNA has also been linked to glycemic values in a type 2 diabetes model [104] and to body mass index (BMI) in patients with metabolic syndrome [16,105]. The enhancer of the zeste homolog 2 (EZH2) gene has been identified as a target of miR-195-5p. This gene encodes a protein of the same name that catalyzes the methylation of histone H3K27me3, altering the expression of its target genes [96]. In GDM-derived HUVECs, this process affects cell proliferation and viability by promoting apoptosis [30]. Additionally, in silico analyses suggest that miR-195-3p expression regulates FGF20, a growth factor involved in embryonic development, cellular growth, morphogenesis, tissue repair, and tumor growth and invasion. MiR-195 also regulates POGLUT3, a gene implicated in muscular dystrophy.

#### 4.2.4. MiR-518d

Fu GD et al. [106] described miR-518d as a member of a cluster of 54 miRNAs located on chromosome 19q13.41. MiR-518d is expressed in both the placenta [30,58] and the serum [87] of women with GDM but is not present in placental extracellular vesicles [24]. Additionally, miR-518d has been identified in the placenta of women who developed preeclampsia, where the fetus exhibited intrauterine growth restriction [107]. Functional studies have shown that miR-518d regulates PPARα, triggering the nuclear transport of NF-κB and phosphorylation of pathway-associated proteins, leading to an inflammatory response linked to GDM. In GDM placentas, a diet enriched with olive oil prevented the expression of PPARα and the upregulation of miR-518d. Furthermore, the hyperactivity of metalloproteases was reduced in both placental tissue and umbilical cord blood. Regarding in silico analyses, it was demonstrated that the aldose reductase (AR) gene is up-regulated by the miR-518 family. AR is located in tissues that do not require insulin for glucose uptake, such as in the eyes, in the corneal epithelium, lens, and retinal pericytes; in the kidneys, in the podocytes, mesangial cells, and tubular epithelium; and in the peripheral nerves, in the axons and Schwann cells. Under hyperglycemic conditions, AR reduces glucose to sorbitol [108]. The production of sorbitol causes cellular and axonal edema (neuropathies) due to increased intracellular osmotic pressure and the inhibition of sodium/potassium ATPase activity in nerve fibers. These alterations trigger the development of microvascular complications in diabetic patients [109]. Despite these findings, significant limitations remain in understanding the role of miR-518d during GDM-affected fetal development.

#### 4.2.5. MiR-29 Family

The miR-29 family comprises miR-29a, miR-29b, and miR-29c; among these, miR-29a and miR-29b are transcribed from chromosome 7q32.3 [110]. Increased expression of this family has been identified in various tissues with metabolic alterations such as obesity, insulin resistance, and type 2 diabetes (T2D). This family is crucial for the functionality of pancreatic β-cells, where it regulates normal insulin exocytosis. Under stress conditions, such as hyperglycemia, the expression levels of the miR-29 family increase, impairing β-cell function [111]. Other studies have linked increased miR-29 expression to disruptions in glucose transport and the development of diabetic nephropathy (DN) [112]. Sonorense’s group [14] demonstrated that elevated levels of miR-29a-3p were characteristic of women with normal glucose tolerance at the beginning of the study who later developed GDM. Additionally, independent studies reported high serum levels of miR-29a-3p between 18 and 23 weeks of gestation (second trimester) in women with GDM [20], and even associating miR-29a-3p expression with male fetuses. Conversely, other studies propose that miR-29a/b could serve as diagnostic markers in pregnant women. The deregulation of miR-29a-3p, alongside other miRNAs (miR-126-3p, -155-5p, -21-3p, -146b-5p, -210-3p, -222-3p, -223-3p, -517-5p, and -518a-3p), enabled the identification of GDM risk during the first trimester [112]. In the absence of miR-29a-3p, the expression of the INSIG1 gene and glucose availability increase through the upregulation of phosphoenolpyruvate carboxykinase 2 (PCK2) [91]. Toward the end of pregnancy, miR-29a/b deregulation was correlated with preterm delivery in women with GDM and elevated glucose levels [20]. In the placenta, miR-29a-3p was also identified in exosomes from women with GDM [51], although no direct association with the tissue was established. Meanwhile, miR-29b regulates HUVEC cell migration. In silico analyses suggest that the expression of the miR-29 family is up-regulated by DNMT3B (DNA methyltransferase 3 beta), SMAD3, FGF7, and PPARG (peroxisome proliferator-activated receptor gamma). Interestingly, DNMT3B is an enzyme responsible for genome methylation during early life stages in fertilized ova. Mutations in the DNMT3B gene have been associated with facial anomalies. SMAD3 and FGF7 play crucial roles in embryonic development. PPARG regulates glucose homeostasis and adipocyte differentiation; mutations or alterations in this gene are associated with insulin resistance and T2D development (Figure 5). These findings suggest that miR-29 is down-regulated in embryos during GDM, although its biological implications remain unclear.

## 5. Conclusions

We identified the miR-29 family, miR-16-5p, -126-3p, -195-5p, and -518b as a molecular signature in the mother–child binomial affected by GDM. To date, analyses on fetal development are limited. However, in silico analyses suggest that the miR-29 family, miR-16-5p, -126-3p, -195-5p, and -518b may play roles in various embryonic alterations under hyperglycemic conditions, affecting the development of the heart, brain, and kidneys. Therefore, it would be important to analyze this group of miRNAs in future studies in GDM.

## 6. Strengths, Limitations, and Recommendations

One significant limitation was the high heterogeneity in the parameters reported for GDM diagnosis. Including all clinical parameters would enable association or correlation analyses with miRNA expression profiles, supporting the utility of miRNAs as biomarkers for early and late diagnosis and risk assessment in women developing GDM, as well as the potential consequences for neonates.

Many articles included in this review focused on placental analysis due to its critical role in maternal–fetal communication. However, none of the studies integrated these results with fetal development.

Another limitation is the lack of functional analyses for the identified miRNAs. Therefore, it is recommended to identify the signaling pathways regulated or affected by miRNAs in GDM.

## Figures and Tables

**Table 1:** Functional studies of some miRNAs identified in gestational diabetes.

Author Year (Cita)	Cellular Line	Expression	Biological Process	Target Genes
Tryggestad, Vishwanath et al., 2016 [69]	HUVEC/BeWo	miR-130b	Insuline regulation/IGF1	AMPKα1
Zhang, Wang et al., 2020 [88]	HRT-8/Svneo/BeWo	miR-136	Pro-apoptosis	E2F1
Ding, Guo et al., 2018 [11]	HTR-8/SVneo	miR-138-5p	Migration Proliferation	TBL1X
Zhao, Zhao et al., 2020 [93]	HTR-8/SVneo/HEK293/HUVEC	miR-140	Insulin resistance	IR-α, IRS-1, and IGF1R
Zhang, Wu et al., 2022 [89]	HUVEC	miR-140-3p	Antiproliferation, anti-migration, tube formation	IL-18 and IL-1β
Muralimanoharan, Maloyan et al., 2016 [49]	Villous cytotrophoblasts	miR-143	Mitochondrial function Glucose metabolism	PPARγ and PGC1α
Tryggestad, Vishwanath et al., 2016 [69]	HUVEC/BeWo	miR-148a	Insulin signaling pathway/IGF2	AMPKα2
Jiang, Wei et al., 2022 [36]	HTR-8/SVneo	miR-17-5p	Glucose captation	TXNIP NLRP3
Villota, Toledo-Rodriguez et al., 2021 [72]	HUVEC	miR-181a-5p	Compromised barrier integrity	OCCLUDIN
Wang, Wei et al., 2021 [80]	Min6	miR-190b	Antiproliferation Anti-insulin secretion	NKX6-1
Liao, Zhou et al., 2020 [45]	HUVEC	miR-195-5p	Cell viability and proliferation Apoptosis	EZH2
Guan, Cao et al., 2022 [32]	JEG-3	miR-199a-5p	Glucose metabolism	MeCP2 and TRPC3
Guan, Tian et al., 2020 [31]	HTR-8/SVneo	miR-21-5p	Anti-proliferation Infiltration	PPAR-α
Song, Su et al., 2021 [5]	HTR-8/SVneo	miR-22	Glucose metabolism	GLUT1 and HK2
Sun, Tian et al., 2020 [66]	HTR-8/SVneo	miR-29b	Anti-migration Invasion	ING2, ING3, and HIF3A
Zhang, Li et al., 2021 [87]	HTR-8/SVneo	miR-30d-5p	Regulation of glycolysis	RAB8A
Li and Zhuang 2021 [44]	HTR-8/SVneo	miR-345-3p	Anti-apoptosis	BAK1
Song, Cai et al., 2021 [63]	HUVEC	miR-34b-3p	Anti-viability Anti-migration	PDK1
Zhang and Zhao 2021 [13]	HTR-8/SVneo	miR-362-5p	Inhibition proliferation Pro-apoptosis	GSR
Wei, L., et al., 2021 [81]	HRT8/SVneo	miR-373	Insulin pathway, including IRS, PI3K, AKT, and GLUT5	SLC2A5
Li et al., 2015 [12]	HTR-8/SVneo	miR-508-3p	Regulator of EGFR	PIKfyve
Zhao et al., 2014 [92]	HEK-293	miR-518d	Antiproliferation	PPAR-α
Qiu, Liu et al., 2020 [59]	HTR8/SVneo	miR-518d	PPARα-mediated NF-κB pathway	PPARα-
Zhang et al., 2020 [89]	HUVEC	miR-574-3p	Antiproliferation	IL-18 and IL-1β
Wang, Wang et al., 2019 [78]	THP-1	miR-657	Anti-inflammatory	IL-37
Wang, Ma et al., 2021 [77]	THP-1	miR-6869-5p	Induces M2 polarization	Protein Tyrosine Phosphatase Receptor type O (PTPRO)
Song, Su et al., 2021 [5]	HTR-8/Svneo	miR-9	Glucose metabolism	GLUT1 and HK3
Chu, Zhong et al., 2024 [23]	HTR-8/SVneo	miR-942-5p	Regulation of trophoblast cells’ biological function	CEBPA
Li, Wang et al., 2018 [42]	INS-1	miR-96	Regulating PAK1 expression, insulin secretion, and β-cell function	PAK1
Yu, Liu et al., 2021 [86]	HRT-8/SVneo	miR-96-5p	Viability	
Cao, Zhang et al., 2016 [9]	HEK-293T/JEG-3	miR-98	Glucose metabolism	Mecp2

**Table 2:** miRNAs determined in cord blood and amniotic fluid in gestational diabetes mellitus.

Author/Year	Tissue	Up-RT-PCR	Down-RT-PCR
Gomez Ribot, Diaz et al., 2020 [30]	Cord blood umbilical blood/placenta	miR-518d	
Shah, Chernausek et al., 2021 [62]	Cord blood, umbilical blood/placenta	miR-126–3p	miR-148a-3p and miR-29a-3p
Liao, Zhou et al., 2020 [45]	Cord blood Umbilical blood	miR-195-5p	
Yu, Liu et al., 2021 [86]	Serum/placenta		miR-96-5p
Li and Zhuang 2021 [44]	Serum/placenta		miR-345-3p
Peng, Li et al., 2019 [53]	Umbilical vein plasma/CEV	miR-137	
Joshi, Azuma et al., 2020 [10]	Amniotic fluid (second trimester)	mR-7-1-3pa, miR-126-3p, miR-185-5p, miR-302a-3p, miR-1268a, miR-146a-5p, miR-15b-5p, miR-197-3p, miR-199a-3pa, miR-378a-3pa, miR-486-3p, and miR-885-5pa	miR-210-3p, miR-99a-5p, and miR-138-5p

**Table 3:** Comparison of circulation miRNAs determined in the mother’s cord blood, umbilical blood, amni-otic fluid, the placenta, and plasma.

MiRNA	Cord Blood Umbilical	Amniotic Fluid (Second Trimester)	Maternal Plasma	Placenta	Ref.
miR-126-3p	1↑	1↑	1↑	2↑	[6,10,62,69]
miR-518d	1↑		1↑	2↑	[30,58,89]
miR-195-5p	1↑		2↑	1↓	[16,67]
miR-345-3p	1↓			1↓	[44]
miR-96-5p	1↓			1↓	[86]
miR-29a-3p	1↓		1↑/4↓	2↓	[14,25,61,66,68]
miR-137	1↑			1↓	[53,54]
miR-148a-3p	1↓			1↑/1↓	[62,69]
miR-185-5p		1↑	1↑	1↑	[10,57]
miR-197-3p		1↑		1↑	[10,51]
miR-199a-3p		1↑		1↑	[10,31]
miR-138-5p		1↓		1↓	[10,11]

The number before the arrow indicates the quantity of reports found. Arrows indicate ↑over-expression or ↓ under-expression.

## Data Availability

The original contributions presented in this study are included in the article/supplementary material. Further inquiries can be directed to the corresponding author(s).

## Supplementary Materials

The following supporting information can be downloaded at: https://www.mdpi.com/article/10.3390/metabo15060367/s1, Table S1: Clinical parameters of the mothers with gestational diabetes and their neonates included in the reviewed studies. Table S2: Expression profile of circulating miRNAs in women with gestational diabetes mellitus. Table S3: Circulating miRNAs in women with gestational diabetes mellitus. Table S4: miRNAs determined in placenta tissue from women with gestational diabetes mellitus. Refs. [113,114] are cited in the Supplementary Materials.
