# Supplementary material for: Circulating MicroRNAs Associated with Changes in the Placenta and Their Possible Role in the Fetus During Gestational Diabetes Mellitus: A Review
Source: Metabolites. 2025 Jun 3;15(6):367. doi: 10.3390/metabo15060367 (PMC12194830; doi:10.3390/metabo15060367)
Supplement: Supplementary file 1 [file metabolites-15-00367-s001.zip › metabolites-3384045-supplementary.pdf]

Table S1. Clinical parameters of the mothers with gestational diabetes and their neonates included in the reviewed studies.

Author/ Year	Tissue	CTR (n)	GDM (n)	Mother's age (years)	BMI	OGTT 0h (mmol/L)	OGTT 1h (mmol/L)	OGTT 2h (mmol/lL)	SISTOLE (mmHg)	DIASTOLE (mmHg)	FBG (mg/dL or mmol/L)	Gestational age (weeks)	Birth weigh (kg)
(Abdeltawab, Zaki et al. 2021)	Placenta	103	109	29.9 ±6.28	23.5 ±1.03	7.94 ±1.09	11.8 ±1.59	10.1 ±1.14			91.88	24-28	
(Akin, Kasap et al. 2024)	Maternal plasma	30	30										
(Cao, Jia et al. 2017)	Maternal plasma	72	85	26.8 ±3.5	25.1 ±2.8	5.1 ±0.9	11.8 ± 2.4	9.5 ±2.2				25.8 ±2.5	
(Cao, Li et al. 2016)	Placenta	202	193	30.93 ±3.45	22.97 ±3.09	5.40 ±0.72	10.99 ±1.44	9.07 ±1.31	117.23 ±11.34	72.90 ±8.93		38 ±0.91	3.43 ±0.47
(Chen and Yan 2024)	Maternal Plasma					≥ 5.1	≥ 10.0	≥ 8.5			5.02 mmol/L	24–28	
(Chu, Zhong et al. 2024)	Placenta	30	15	29.33	24.1	5.2	9.74	8.34			5.2 mmol/L	39	3.503
(Dai, Zhu et al. 2020)	Maternal serum	60	67										
(Deng, Huang et al. 2020)	Maternal serum	55	68	32.65 ±4.63	23.06 ±3.56				115.62 ±9.29	74.08 ±6.92		39.09 ±1.11	
(Ding, Guo et al. 2018)	Placenta	26	28	32.88 ±1.043	22.46 ±3.19	4.48 ± 0.37	10.94 ±1.12	9.73 ±0.75			91.88	38-40	3.386 ±0.311
(Ding, Shen et al. 2022)	Maternal serum	8	11	28.17 ±4.070		5.16 ±0.499	9.15 ±0.33	7.33 ±0.859	115.83 ±18.713	76.00 ±12.458	5.16 ±0.499 mmol/L		
(Feng, Qu et al. 2020)	Maternal serum	12	12										3787.4
(Filardi, Catanzaro et al. 2022)	Maternal serum	15	15	34.7 ±4.9	26.3 ±4.6							36.6 ±1.2	
(Ghaneialvar, Mohseni et al. 2024)	Maternal serum	40	40						4.36 ± 82.22	13.71 ± 124.33	22.5 ±139.50		

(Gillet, Ouellet et al. 2019)	Maternal serum/ EV	46	23	29.8 (5.3)	28.2 ±7.2				120.6 (15.2)	76.6 (9.1)		38.4 (2.1)	3.424 ±0.458
(Gomez Ribot, Diaz et al. 2020)	Cord blood umbilical/Placenta	17	33	29.6 ± 1.6							89.0 ±3.6	24-28	3.205 ±0.113
(Guan, Cao et al. 2022)	Maternal serum	158	137	30.93 ±3.4	22.97±3.1			9.07 ±1.3					
(Guan, Tian et al. 2020)	Placenta	158	137	30.94 ±3.45	22.97 ±3.09		194.39 ±26.11	163.35 ±23.59	117.24 ±11.34	72.90 ±8.93	97.07 ±12.79	38.88 ±0.91	
(He, Bai et al. 2017)	Maternal peripheral venous blood	20	20										
(He, Wang et al. 2024)	Maternal peripheral venous	60	60	29.38 ±3.12								35.27 ±3.18	
(Hromadnikova, Kotlabova et al. 2022)	Placenta	121	80										
(Hu, Li et al. 2021)	Placenta	35	35	34 ±4	28.5 ±4.1								
(Hua, Li et al. 2021)	Maternal white blood cells	38	30	30.27 ±6.38	27.82							37-39	3.54 ±0.58
(Jamalpour, Zain et al. 2022)	Maternal serum	24	24	30.58 ± 3.844	21.11 ± 3.086						7.5 (6–8.5) mmol/L		3.270 (3.1–3.5)
(Ji, Zhang et al. 2020)	Maternal blood/placenta	20	20										
(Jiang, Wei et al. 2022)	Placenta	16	16	32.06 ±2.98	21.75 ±1.76	5.14 ±0.53	10.58 ±1.30	8.84 ±1.10				38.13 ±0.89	3.391 ±0.2956
(Joshi, Azuma et al. 2020)	Amniotic Fluid	20	20	37.4 ±3.6									
(Juchnicka, Kuźmicki et al. 2022)	Maternal peripheral venous blood	24	24			158 (148-165)	169 (136.5-184)	125.5 (111.5-166)				25 (25-26)	
(Kunysz, Cieśla et al. 2024)	Maternal Plasma	30	42	33.5	29.75						77	36.5	3.4

(Lamadrid-Romero, Solís et al. 2018)	Maternal serum	75	67										
(Légaré, Desgagné et al. 2022)	Maternal serum	380	56	29.71 ±5.6	28.16 ±7.3	4.66 ±0.6	9.85 ±1.4	8.24 ±1.3				9.51 ±2.8	
(Légaré, Desgagné et al. 2024)	Maternal serum	58	484	31.3 ±4.4 (22.0–45.0)	26.4 ±6.6 (18.1–47.2)	4.7 ±0.5 (3.7–6.6)	8.9 ±1.6 (4.7–12.3)	7.1 ±1.4 (3.8–11.5)				27.3 ±1.4 (24.4–30.0)	
(Li and Zhuang 2021)	Serum/Placenta	30	30	24-39									
(Li, Monazzam et al. 2021)	Placenta	30	30	24-39								24- 28	
(Li, Song et al. 2015)	Placenta	15	15	21-37							91.88		4.042 ±0.104
(Li, Wang et al. 2018)	Placenta	3	3										
(Liao, Zhou et al. 2020)	Maternal serum	25	25	25-45									
(Lin, Chen et al. 2024)	Maternal serum	5	5	29.60 ±2.074	21.800 ±2.035	4.626 ±0.481	10.162 ±1.036	8.210 ±1.967					
(Liu, Feng et al. 2023)	Maternal serum	30	30	32.02 ±2.18	24.54±1.86								2.54 ±1.86
(Liu, Zhang et al. 2021)	Cord blood, umbilical/placenta	78	110	33.118 ±3.402	23.466 ±3.326							25.591 ±1.757	
(Marei and Gabr Youssef 2020)	Maternal serum	20	20	29.02 ±2.244	32.21 ±4.06				118.5 ±16.66	75.5 ±9.30	95	36.1 ±3.47	3.95 ±0.416
(Muralimanoharan, Maloyan et al. 2016)	Placental exosomes	6	6	32 ±1	27.8 ±1.5						95.6 ±3.1	39.1 ±0.5	3.511 ±0.116
(Nair, Guanzon et al. 2021)	Maternal serum	8	29	36.9 ±3.02	23.5 ±5.46	4.58 ± 0.53	11.1 ±1.15	10.0 ±1.45				24.4 ±1.25	3.154 ±1.00

(Nair, Jayabalan et al. 2018)	Placental	12	12	30.5 ±4.19	28.17 ± 1.29	5.13 ± 0.58	10.22 ±2.55*	9.77 ±0.90**				>37	
(Niu, Yu et al. 2022)	Maternal peripheral venous blood	85	85	26.87 ±3.40	29.82 ±2.40						5.76 ± 0.43 mmol/L	38.66 ±1.70	3.404.71 ±0.420
(Peng, Li et al. 2018)	Umbilical vein plasma/CEV	12	11	29.91 ±1.385	27.71 ±0.6067	5.29 ±0.1053	10.27 ±0.3441	8.57 ±0.1954			91.88	24-28	
(Pfeiffer, Sánchez-Lechuga et al. 2020)	Cord blood umbilical	29	31	31.9 ±1.8	22,5 ±1.8				106.9 ±12.3	64 ±9	92.5 ±13	39.1 ±1.3	3.202 ±0.589
(Pheiffer, Dias et al. 2018)	Maternal serum	28	53	29.5 ±6.2	28.1 (23.9–31.3)						5.6 (5.1–6.1) mmol/L		
(Qi and Wang 2019)	Maternal blood/placenta		156										
(Qiu, Liu et al. 2020)	Placenta	60	60									36-42	
(Qiu, Shangguan et al. 2020)	Maternal Plasma	32	44										
(Sebastiani , Guarino et al. 2017)	Maternal serum	4	4	35.00 ±3.91	21.75 ± 1.11						80.00 ± 2.16		
(Serati, Novielli et al. 2023)	Maternal serum	7	6	33.33 ±6.83	32.55 ±2.23*	84.60 ±6.23	125.40 ±36.31	109.40 ±30.18				39.00 ±0.42	3.415 ±0.386
(Shah, Chernausek et al. 2021)	Maternal serum	47	23	32.1 ±5.5	35.7 ±7.8							38.9 ±0.9	3.70 ±0.55
(Song, Cai et al. 2021)	Placenta	27	20										
(Song, Su et al. 2021)	Placenta	20	31	25.8 ±3.6	23.8 ±2.3	4.6 ±0.5	10.9 ±0.7	8.9 ±0.4			91.88	38-39	3.628 ±0.3439
(Sørensen, van Poppel et al. 2021)	Serum/Placenta	41	82	32.7 ±4	33.3			6.85			5.05 mmol/L		

(Sørensen, van Poppel et al. 2022)	Placenta	41	41	33.3 (31.7-35.9)	105.6±8.8	4.9 (4.6-5.2)	7.2 (6.2-8.3)	6.4 (5.7-7.4)			4.9 (4.6-5.2) mmol/L	24–28	3.557 ±0.496
(Stirm, Huypens et al. 2018)	Maternal serum												
(Sun, Tian et al. 2020)	Maternal serum	202	204	30.91 ±0.38	22.95 ±3.11	5.77 ± 5.49	10.92 ±1.49	9.07 ±1.52	117.47 ±11.29	73.22 ±9.02	91.88	>37	3.35 ±0.59
(Tagoma, Alnek et al. 2018)	Maternal serum												
(Toljic, Nikolic et al. 2024)	Maternal plasma	34	31										
(Tryggestad, Vishwanath et al. 2016)	Placenta	12	7	27.6 ±4.9	34.5 ±7.8							>37	3.61 ±0.4
(Tu, Wang et al. 2020)	Placenta	73	76	27.02 ±3.17								24-28	
(Valerio, Barabash et al. 2022)	Maternal serum	236	77	34.9 ±4.6			159.4 ±33.3	135.1 ±31.3	108 ±11	65 ± 8	92.6 ±6.7	24 − 28	
(Villota, Toledo-Rodriguez et al. 2021)	Maternal serum	21	11	31.83	32.38							37-38	3.62
(Wander, Boyko et al. 2017)	Maternal plasma	80	36	34.3 ±3.6	25.5 ±6.7							15.1 ±2.9	
(Wang, Ma et al. 2021)	Maternal plasma	23	26	30.6 ±4.4		5.1 ±1.0	10.8 ± 1.7	8.5 ±1.4	119.3 ±11.1	71.9 ±7.2	88.46	38-40	3.708 ±0.1102
(Wang, Ma et al. 2021)	Placenta (2019)	48	53	29.8 ±0.4	26.4 ±0.4	>5.1	>10.0	>8.5			5.1 mmol/L		
(Wang, Pan et al. 2020)	Placenta	5	5										
(Wang, Wang et al. 2019)	Placental exosomes	46	48	29.86 ±0.94		4.77 ± 0.22	9.59 ±0.66	8.93 ±0.54	124.01 ± 5.98	78.61 ±5.77	85.94		3.590 ±0.869

(Wang, Wang et al. 2019)	Maternal serum	100	100		28.41 ±2.18						79.48 ±2.95	24-28	
(Wang, Wang et al. 2020)	Maternal serum	102	102	29.8 ±3.2	28.3 ±4.8		10.2 ±0.3	8.3 ±0.2			91.88 mg/dL	27.0 ±1.6	
(Wei, Cao et al. 2021)	Placenta	75	75		25.17 ± 1. 65							27.54 ±2.8	
(Wen and Bai 2021)	Placenta	48	32	32.71 ±5.26								28.33 ±2.81	
(Xiao, Ding et al. 2020)	Maternal serum	10	30			≥5.1	≥10	≥8.5			92.06		
(Xu, Bian et al. 2017)	Maternal Plasma	3	3										
(Ye, Wang et al. 2022)	Maternal Serum	24	113	33.53 ±4.16	22.41 ± 3.25	25.49 ±1.73	24.64 ±3.18				4.68 ±0.45 mmol/L	14.92 ±3.72	
(Yoffe, Polsky et al. 2019)	Maternal plasma			34.0 (32.5–37.5)	28.6 (20.4–31.1)		9.0 (8.2–10.4)	7.3 (6.1–8.5)			92.06	10 (10.0–10.6)	3.555 (3.060–4.016)
(Yu, Liu et al. 2021)	Maternal plasma	123	123	31.23±2.31		5.57±0.49	10.25±0.53	8.48±0.22			5.57±0.49 mmol/L	39.35±3.81	
(Zhang and Chen 2020)	Placenta	61	57	32.39 ±4.60	30.58±3.6 0	5.49 ±0.85	11.29±1.34	9.71 ±1.64	127.25 ±10.87	81.17 ±9.64		37-41	
(Zhang, Li et al. 2021)	Serum/Placenta	196	166		22.44 ±3.08				116.67 ±11.47	73.04 ±8.70	95.3 ±11.53		3.378 ±0.596
(Zhang, Li et al. 2021)	Maternal serum	40	40	31.72 ±2.15	27.44 ± 3.83	5.58 ±0.52	10.32 ±0.48	8.39 ±0.26			5.58 ±0.52 mmol/L	39.47 ±2.02	3.42 ±0.25
(Zhang, Wang et al. 2020)	Maternal peripheral venous blood	30	30										
(Zhang, Wu et al. 2022)	Placenta	30	30	35.10 ±3.55							10.60 ±1.10 mmol/L	38.50 ±0.68	
(Zhang, Ye et al. 2023)	Placental exosomes	30	30	29.23 ± 0.5 944	27.28 ± 0. 4066				118.90 ±1.0 690	74.90 ±1.1930	5.03 ± 0.1811 mmol/L	275.50 ±1.217 days	3.3 ±0.7439
(Zhang, Zhang et al. 2020)	Placenta	58	112	31.59 ±3.93	23.35 ±3.56						117.28±6. 13	24.85 ±1.69	

(Zhao, Dong et al. 2011)	Maternal serum	12	14	28.79 ±2.21	21.44 ±1.70						5.30 ±0.90 mmol/L	17.40 ±0.70	
(Zhao, Zhang et al. 2014)	Placenta	40	40	30.55 ±3.30	23.54 ±3.46							39.61 ±0.76	4.007 ±0.489
(Zhao, Zhao et al. 2020)	Maternal peripheral venous blood /placenta	21	12		<25							38-41	
(Zhou, Xiang et al. 2019)	Maternal serum	50	108	30.89 ±3.45	22.82 ± 2.57						113.68 ± 5.58	25.20 ±1.23	

Abbreviations: CTR= Control, GDM= Gestational diabetes mellitus, OGTT= Oral glucose tolerance test; FBG=Fasting blood glucose, BMI = Body mass index.

Table S2. Expression profile of circulating miRNAs in women with gestational diabetes mellitus.

Author Year	Pregnancy trimester	Methodology	Up-miR	Down-miR	Validation by qPCR
(Zhao, Dong et al. 2011)	Second	Microarray		miR-132, miR-29a y miR-222	**miR-29a, miR-222**
(Zhu, Tian et al. 2015)	Second	DESeq2	miR-16-5p, miR-17-5p, miR-19a-3p, miR-19b-3p, miR-21-5p, miR-20a-5p, miR-92a-3p, miR-126-3p, miR-151a-5p, miR-234-3p, miR-185-5p, miR-484,	Let-7d-5p, Let-7b-5p, Let-7c, Let-7i-5p, miR-15a-5p, miR-26b-5p, miR-23b-3p, miR-30a-5p, miR-92b-3p, miR-99a-5p, miR-107, miR-137, miR-146a-5p, miR-487b, miR-125a-5p, miR-221-3p, miR-3591-3p, miR-497-5p, miR-483-5p, miR-885-5p.	**miR-16-5p**, miR-17-5p, miR-19a-3p, miR-19b-3p, miR-20a-5p
(Xu, Bian et al. 2017)	Second	Microarray	miR-23b-3p, miR-27a-3p, miR-126-3p, miR-126-5p, miR-20b-5p, miR-142-3p, miR-181c-5p, miR-181d, miR-181a-5p, miR-181b-5p, miR-199b-3p, miR-5704, miR-199b-5p, miR-4791, miR-374c-3p, miR-452-5p, miR-454-3p, miR-455-3p, miR-503, miR-634, miR-1255b-2-3p, miR-377-3p, miR-3591-3p, miR-3182, miR-4770, miR-4694-5p, miR-H5-3P miR-5006-3p	miR-135a-3p, miR-141-3p, miR-200c-3p, miR-1587, miR-3162-3p, miR-3621, miR-4505, miR-4508, miR-4488, miR-4787-3p, miR-4769-3p, miR-4507, miR-4725-3p, miR-US25-1-3P, miR-BART19-3P	miR-503
(Sebastiani, Guarino et al. 2017)	Second	Microarray	miR-330-3p, miR-483-5p	miR-532-3p, miR-548c-3p,	miR-330-3p, miR-548c
(Stirm, Huypens et al. 2018)	Second	RNA-seq	Let-7a-5p, Let-7f-5p, Let-7g-5p, Let7e-5p, Let7i-5p, miR-7c-5p, miR-15a-5p, miR-17-5p, miR-17-3p, miR-18a-5p, miR-19a-3p, miR-19-3p, miR-22-3p, miR-93-5p, miR-96-5p, miR-103a-3p, miR-103b, miR-107, miR-143-3p, miR-142-5p, miR-145-5p, miR-148a-3p, miR-199a-3p, miR-199b-3p, miR-340-5p, miR-451a, miR-660-5p, miR-1307-3p, miR-106b-5p.		Let-7g-5p, miR-19a-3p, miR-142-5p, miR-143-3p, miR-340-5p
(Tagoma, Alnek et al. 2018)	Second	Microarray	Let-7e-5p, Let-7g-5p, miR-23b-3p, miR-30b-5p, miR-30c-5p, miR-30d-5p, miR-100-5p, miR-101-3p, miR-146a-5p, miR-18a-5p, miR-195-5p, miR-222-3p, miR-342-3p, miR-423-5p, miR-92a-3p.		miR-92a-3p, miR-30d-5p, **miR-195-5p.**
(Nair, Guanzon et al. 2021)	First, second and third	RNA-seq	101 miRNAs (no descritos)		‹et-7i-5p, miR-10a-5p, miR-16-2-3p, **miR-16-5p,** miR-92a-3p, miR-92b-3p, miR-151b, miR-423-5p, miR-1910-5p
(Wang, Li et al. 2021)	Second	Microarray	Let-7g-5p, miR-22-5p, miR-28b-5p, miR-122-5p,miR-145-5p, miR-572, miR-615-3p, miR-630, miR-1280, miR-371b-5p, miR-1237-3p, miR-1227-5p, miR-1587, miR-1716-3p, miR-1728-5p, miR-4443, miR-4644, miR-4721, miR-4707-5p, miR-4780-3p, miR-4787-5p, miR-5703, miR-6075, miR-6125, miR-6123, miR-6752-3p, miR-6779-5p, miR-6851-3p, miR-6870-3p, miR-7704.	miR-32-3p, miR-211-3p, miR-328-3p, miR-300, miR-484, miR-595, miR-574-5p, miR-809, miR-819-5p, miR-1248-3p, miR-1224-5p, miR-1972, miR-2135b, miR-2278, miR-3661, miR-4285, miR-4695-5p, miR-4787-3p, miR-4728-3p, miR-4648-5p, miR-4651, miR-4438b-5p, miR-4701-3p, miR-5825-5p, miR-8071, miR-8768-5p,	miR-574-5p, miR-3135b

(Légaré, Desgagné et al. 2022)	First	RNA-seq	miR-4477b, miR-423-5p, miR-501-3p, miR-3160-3p	miR-10a-3p, miR-141-3p, miR-149-5p, miR-154-3p, miR-196a-5p, miR-218-5p, miR-200c-3p, miR-338-3p, miR-376, miR-373-3pc, miR-512-3p, miR-517-5p, miR-517a-3p|miR-517b-3p, miR-519c-3pb, miR-520a-3p, miR-548av-5p|miR-548k, miR-524-5p, miR-516b-5p, miR-577, miR-548j-3p, miR-518a-5p|miR-527, miR-518e-5p|miR-519a-5p|miR-519b-5p|miR-519c-5p|miR-522-5p|miR-523-5p, miR-1285-5p, miR-1277-5p, miR-1277-3p, miR-1323, miR-4433b-3p, miR-1256.	Let7a-3p, miR-218-5p, miR-517a-3p|miR-517b-3p
(Juchnicka, Kuźmicki et al. 2022)	First	NanoString technology	miR-16-5p, miR-142-3p, miR-144-3p,		**miR-16-5p,** miR-142-3p, miR-144-3p, miR-320e
(Jamalpour, Zain et al. 2022)	First, second and third	Microarray	first trimester= miR-193a, miR-21, miR-23a, miR-361.<br><br>second trimester= Let7-i, miR-126, miR-129.<br><br>Third trimester= Let7e, miR-107, miR-361, miR-370	First trimester= miR-130a.<br><br>Second trimester= miR-125, miR-129–2, miR-130a, miR-34, miR-375.<br><br>Third trimester= miR-125, miR-129, miR-130a.	miR-21, miR-23a, miR-193a,miR-130a, miR-361
(Filardi, Catanzaro et al. 2022)	First, second and third	NanoString nCounter Human v3 miRNA expression assay	miR-222-3p, miR-302d-3p, miR-874-3p, miR-941,	miR-197-5p, miR-337-5p, miR-323a-3p, miR-409-3p, miR-432-5p, miR-625-5p, miR-6721-5p.	**miR-222-3p,** miR-409-3p
(Ye, Wang et al. 2022)	Second	Small-RNA NGS	miR-1-3p, miR-16-2-3p, miR-19b-3p, miR-186-5p, miR-423-5p.	Let-7c-5p, Let-7f-5p, miR－21-5p, miR-22-5p, miR－26b-5p, miR-27b-3p, miR-99a-5p, miR-100-5p, miR-122-5p, miR-125b-5p, miR-148a-3p, miR-151a-3p, miR-192-5p, miR-194-5p, miR-375-3p, miR-1290, miR-1246.	miR-423-5p, miR-122-5p,
(Ding, Shen et al. 2022)	Second	RNA-seq	Let-7d-3p, miR-151a-3p, miR-181b-3p, miR-326, miR-345-5p, miR-378a-5p, miR-425-3p, miR-484, miR-486-3p, miR-486-5p, miR-500a-3p, miR-652-3p, miR-11401, miR-1908-5p, miR-3127-3p, miR-3177-3p, miR-4664-3p, miR-6852-5p, miR-6515-5p, miR-7976.	Let-7f-5p, miR-7-5p, miR-9-5p, miR-19b-3p, **miR-29b-1-5p, miR-29b-3p,** miR-34c-5p, miR-125a-5p, miR-142-3p, miR-155-5p, miR-181d-5p, miR-196b-5p, miR-200c-3p, miR-212-5p, miR-301a-3p, miR-365a-5p, miR-375-3p, miR-409-5p, miR-432-5p, miR-454-3p, miR-493-5p, miR-508-3p, miR-542-3p, miR-543, miR-516b-5p, miR-548e-3p, miR-582-5p, miR-664b-5p, miR-889-3p, miR-1255a.	
(Chen and Yan 2024)	Second	RNA-seq	miR-146a-5p, miR-548, miR-1285-5p, miR-1911-5p, miR-4726-5p, miR-4690-5p, miR-6831-3p.	miR-146a-3p, miR-146b-3p	miR-146a-3p
(Serati, Novielli et al. 2023)	Second	Microarray	Let-7b-5p, miR-21, miR-186-5p, miR-320d, miR-320c, miR-574-3p,	Let-7f-5p, miR-21-5p, miR-27a-3p, miR-30e-3p, miR-33a-5p, miR-324-5p, miR-142-3p-3, miR-339-5p.	miR-454-3p
(Liu, Feng et al. 2023)	Second	Microarray	miR-222, miR-429, miR-520,	miR-143-3p, miR-3135b, miR-574-5p.	**miR-143-3p**

(Légaré, Desgagné et al. 2024)	First	DESeq2	miR-143-3p	miR-143-3p, miR-145-3p, miR-155-5p, miR-512-3p, miR-515-5p, miR-515-3p, miR-516b-5p, miR-516a-5p, miR-517-5p, miR-517c-3p, miR-518-3p, miR-519-3p, miR-519d-5p, miR-520-3p, miR-524-5p, miR-524-3p, miR-525-5p, miR-526b-5p, miR-1283, miR-1323, miR-3191-3p, miR-518-5p|miR-519-5p|miR-519-5p|miR-519-5p|miR-522-5p|miR-523-5p, miR-517-3p|miR-517-3p, miR-518a-5p|miR-527	**miR-143-3p**
(Lin, Chen et al. 2024)	Third	RNA-seq	miR-96, miR-122-5p, miR-3127-5p, miR-5193, miR-5003-3p, miR-6734-5p.	miR-10395-3p	

Table S3. Circulating miRNAs in women with gestational diabetes mellitus.

Author year	Tissue	Up-RT-PCR	Down-RT-PCR
(Qi and Wang 2019)	Maternal blood/placenta	miR-185	
(Ji, Zhang et al. 2020)	Maternal blood/placenta		miR-193b

(He, Bai et al. 2017)	Maternal peripheral venous blood		miR-494
(Xiao, Ding et al. 2020)	Maternal peripheral venous blood	miR-330-3p	
(Zhang and Chen 2020)	Maternal peripheral venous blood	miR-770-5p	
(Hromadnikova, Kotlabova et al. 2022)	Maternal peripheral venous blood	miR-1-3p, miR-20a-5p, miR-20b-5p, miR-23a-3p, miR-100-5p, miR-125b-5p, **miR-126-3p,** miR-181a -5p, **miR-195-5p**, miR-499a-5p y miR-574-3p.	
(Niu, Yu et al. 2022)	Maternal peripheral venous blood	miR-221, miR-320	
(He, Wang et al. 2024)	Maternal peripheral venous blood	**miR-122**	
(Wander, Boyko et al. 2017)	Maternal plasma	miR-21-3p, **miR-29a-3p**, miR-126-3p, miR-155-5p, miR-146b-5p, miR-210-3p, **miR-222-3p**, miR-223-3p, miR-517-5p, miR-518a-3p.	
(Cao, Jia et al. 2017)	Maternal plasma	**miR-16-5p,** miR-17-5p, miR-20a-5p	
(Yoffe, Polsky et al. 2019)	Maternal plasma	miR-223, miR-23a	
(Wen and Bai 2021)	Maternal plasma	miR-520h	
(Kunysz, Cieśla et al. 2024)	Maternal plasma	**miR-16-5p,** 21-5p, 222-5p	
(Toljic, Nikolic et al. 2024)	Maternal plasma	miR-17, miR-181a	**miR-29a**
(Qu, Liu et al. 2022)	Maternal plasma		miR-451a
(Lamadrid-Romero, Solís et al. 2018)	Maternal serum	miR-125b-5p, miR-183-5p, miR-200b-3p, miR-1290,	
(Pheiffer, Dias et al. 2018)	Maternal serum		**miR-16-5p**, miR-17-5p, miR-19a-3p, miR-19b-3p, miR-20a-5p, **miR-29a-3p, miR-132.3p, miR-222-3p**
(Zhou, Xiang et al. 2019)	Maternal serum		miR-132
(Dai, Zhu et al. 2020)	Maternal serum	miR-2467	
(Deng, Huang et al. 2020)	Maternal serum		**miR-29a, miR-29b**
(Pfeiffer, Sánchez-Lechuga et al. 2020)	Maternal serum	miR-330-3p	
(Feng, Qu et al. 2020)	Maternal serum	miR-33a-5p	
(Tu, Wang et al. 2020)	Maternal serum	miR-409-5p	

(Abdeltawab, Zaki et al. 2021)	Maternal serum	miR-223	
(Liu, Zhang et al. 2021)	Maternal serum	miR-1323	
(Song, Su et al. 2021)	Maternal serum	**miR-16-5p**, **miR-29a-3p,** miR-134- 5p, **miR-122-5p**,	
(Hua, Li et al. 2021)	Maternal serum	miR-377-3p	
(Liu, Zhang et al. 2021)	Maternal serum	miR-1323	
(Valerio, Barabash et al. 2022)	Maternal serum	**miR-222-3p**, miR-132	**miR-29a-3p**
(Sørensen, van Poppel et al. 2022)	Maternal serum	**miR-29a-3p**; miR-134-5p	
(Ghaneialvar, Mohseni et al. 2024)	Maternal serum	miR-26a, miR-26b	
(Wang, Zhang et al. 2019)	Maternal serum	miR-19a, miR-19b	
(Wang, Pan et al. 2020)	Maternal serum	**miR-195-5p**	
(Qi and Wang 2019)	Maternal serum/ placenta	miR-185	
(Gillet, Ouellet et al. 2019)	Maternal serum EV	miR-517-5pa, miR-517a-3pa, miR-518ba, miR-520ha, miR-525-5pa, miR-1323a, miR-136-5pb, miR-342-3p, miR-376c-5pb, miR-494-3pb, **miR-29a-3p**, **miR-29b-3p**, miR-122-5p, miR-132-3p, miR-182-3p, miR-210-3p, miR-483-3p	
(Hu, Mu et al. 2021)	Maternal white blood cells		miR-4646

Table S4. miRNAs determined in placenta tissue from women with gestational diabetes mellitus.

**Author**<br>**Year**	**Methodology**	**Up-miR**	**Down-miR**
(Shah, Chernausek et al. 2021)	RT-qPCR	miR-126-3p	Let-7a-5p, miR-130b-3p, miR-148a-3p, miR-29a-3p

(Li, Wang et al. 2018)	RT-qPCR		miR-96
(Akin, Kasap et al. 2024)	RT-qPCR	miR-139-5p	miR-129-5p
(Zhang, Zhang et al. 2020)	RNA-seq	miR-128-3p, miR-1246	miR-22-5p, miR-27a-5p, **miR-29c-5p, miR-29b-1-5p**, miR-30b-3p, miR-30c-1-3p, miR-34a-5p, miR-141-5p, miR-140-3p, **miR-195-5p,** miR-331-5p, miR-320a-3p, miR-362-5p, miR-362-3p, miR-375-3p, miR-500a-3p, miR-501-3p, miR-502-3p, miR-518e-5p, miR-518a-3p, miR-518b, miR-519d-3p, miR-519a-2-5p, miR-526a-3p, miR-520f-5p, miR-548ai, miR-548i, miR-561-5p, miR-574-5p, miR-574-3p, miR-584-5p, miR-767-5p, miR-766-3p, miR-874-3p, miR-1296-5p, miR-3196.
(Jiang, Wei et al. 2022)	RNA-seq		miR-17-5p
(Li, Yuan et al. 2022)	RNA-seq		miR-22, miR-372
(Li, Song et al. 2015)	Microarray	miR-10a, miR-508-3p, miR-148b.	Let-7d, miR-7, miR-9, miR-15b, miR-25, miR-27a, miR-30d, miR-30d, miR-33a-5p, miR-92a, miR-137, miR-192, miR-214, miR-362-5p, miR-370, miR-492-5p, miR-502-5p, miR-516-3p, miR-519, miR-551b, miR-597, miR-758, miR-889, miR-942, miR-1290, miR-1225-3p.
(Sun, Tian et al. 2020)	RT-qPCR		miR-29b
(Zhang, Li et al. 2021)	RT-qPCR		miR-30d-5p
(Li and Zhuang 2021)	RT-qPCR		miR-345-3p
(Ding, Guo et al. 2018)	RNA-seq	miR-187-3p, miR-202-5p, miR-205-3p, miR-767-3p, miR-4763-5p, miR-5096, miR-6824-5p, miR-6844, miR-1273h-5p.	miR-95-3p, miR-138-1-3p, miR-138-5p, miR-210-5p, miR-216a-3p, miR-203b-3p, miR-548a-5p, miR-718, miR-1208, miR-1291, miR-1469, miR-2114-5p, miR-3158-3p, miR-3130-3p, miR-3914, miR-4500, miR-4488, miR-4685-3p, miR-4792, miR-4732-3p, miR-4492, miR-6892-3p, miR-7975.
(Zhang, Zhang et al. 2020)	RNA-seq	miR-15b-5p, **miR-16-5p**, miR-21-5p, miR-32-5p, miR-142-3p, miR-144-5p, miR-451a, miR-512-5p	miR-1-3p, miR-125b-5p, miR-151a-5p, miR-517-5p, miR-409-3p, miR-543,
(Wang, Ma et al. 2021)	RT-qPCR		miR-6869-5p
(Song, Su et al. 2021)	RT-qPCR		miR-9, miR-22

(Chu, Zhong et al. 2024)	RT-qPCR		miR-942-5p
(Zhang and Zhao 2021)	RT-qPCR		miR-362-5p
(Zhao, Zhang et al. 2014)	RT-qPCR	**miR-518d**	
(Cao, Zhang et al. 2016)	RT-qPCR	miR-98	
(Tryggestad, Vishwanath et al. 2016)	Microarray	Let-7g-5p, Let-7a-5p, **miR-29b-3p,** miR-30c-5p, miR-93-5p, **miR-126-3p**, miR-130b-3p, miR-148a-3p, miR-191-5p, miR-221-3p, miR-216a-5p, miR-320a, miR-452-5p, miR-485-3p, miR-489-3p, miR-513a-5p, miR-542-5p, miR-548a, miR-610, miR-616-5p, miR-1273f, miR-1246, miR-1273d, miR-1973, miR-2392, miR-4710, miR-4786-3p, miR-4458, miR-4753-5p, miR-5006-5p, miR-5096, miR-642a-3p.	
(Zhao, Zhao et al. 2020)	RT-qPCR	miR-140	
(Villota, Toledo-Rodriguez et al. 2021)	RT-qPCR	miR-181a-5p	
(Guan, Tian et al. 2020)	RT-qPCR	miR-199a	
(Jiang, Wei et al. 2022)	RT-qPCR	miR-17-5p	
(Qiu, Liu et al. 2020)	RT-PCR	**miR-518d**	
(Wang, Wei et al. 2021)	RT-PCR	miR-190b	
(Zhang, Wang et al. 2020)	RT-qPCR	miR-136	
(Song, Cai et al. 2021)	RT-qPCR	miR-34b-3p	
(Guan, Tian et al. 2020)	RT-qPCR		miR-21-5p
(Nair, Jayabalan et al. 2018)	RNA-seq	miR-22-3p, miR-99b-5p, miR-125a-3p, miR-186-5p, miR-197-3p, miR-224-5p, miR-433-3p, miR-423-3p miR-584-5p.	miR-140-3p, miR-145-3p, miR-144-3p, miR-208a-3p, miR-203a-3b, miR-335-5p, miR-411-5p, miR-451a, miR-369-3p, miR-483-3p, miR-574-3p, miR-550a-3-3p, miR-6795-5p.

(Muralimanoharan, Maloyan et al. 2016)	RT-qPCR		miR-143
(Wang, Wang et al. 2019)	RT-qPCR	miR-657	
(Zhang, Ye et al. 2023)	RT-qPCR	miR-135a-5p	
(Marei and Gabr Youssef 2020)	RT-qPCR	miR-16	miR-221
(Li and Zhuang 2021)	RT-qPCR		miR-345-3p
(Yu, Liu et al. 2021)	RT-qPCR		miR-96-5p

References

1. **Abdeltawab, A., M. Zaki, Y. Abdeldayem, A. Mohamed and S. Zaied (2021). "Circulating micro RNA-223 and angiopoietin-like protein 8 as biomarkers of gestational diabetes mellitus." British Journal of Biomedical Science 78(1): 12-17.**
2. **Akin, M. N., B. Kasap, F. Pirincci, B. Sezgin, C. Ozdemir, A. D. Bilgic, Y. Aftabi and T. G. Edgunlu (2024). "Changes of miR-139-5p, TGFB1, and COL1A1 in the placental tissue of cases with gestational diabetes mellitus." Gene 897: 148061.**
3. **Akin, M. N., B. Kasap, F. Pirincci, B. Sezgin, C. Ozdemir, A. Demirtas Bilgic, Y. Aftabi and T. Gokdogan Edgunlu (2024). "Changes of miR-139-5p, TGFB1, and COL1A1 in the placental tissue of cases with gestational diabetes mellitus." Gene 897: 148061.**

4. Cao, J.-L., L. Zhang, J. Li, S. Tian, X.-D. Lv, X.-Q. Wang, X. Su, Y. Li, Y. Hu and X. Ma (2016). "Up-regulation of miR-98 and unraveling regulatory mechanisms in gestational diabetes mellitus." Scientific Reports 6(1): 32268.
5. Cao, Y. L., Y. J. Jia, B. H. Xing, D. D. Shi and X. J. Dong (2017). "Plasma microRNA-16-5p, -17-5p and -20a-5p: Novel diagnostic biomarkers for gestational diabetes mellitus." J Obstet Gynaecol Res 43(6): 974-981.
6. Chen, M. and J. Yan (2024). "A preliminary integrated analysis of miRNA-mRNA expression profiles reveals a role of miR-146a-3p/TRAF6 in plasma from gestational diabetes mellitus patients." Ginekol Pol 95(8): 627-635.
7. Chu, Q., X. Zhong, Y. Lu and Y. Xu (2024). "miR-942-5p Regulates Proliferation, Invasion and EMT of Trophoblast Cells in Gestational Diabetes by Targeting the CEBPA." Altern Ther Health Med 30(9): 312-318.
8. Dai, S., X. Zhu and H. Xia (2020). "MiR-2467 is a Potential Marker for Prediction of Gestational Diabetes Mellitus in Pregnancy." Clin Lab 66(10).
9. Deng, L., Y. Huang, L. Li, H. Chen and J. Su (2020). "Serum miR-29a/b expression in gestational diabetes mellitus and its influence on prognosis evaluation." Journal of International Medical Research 48(9): 0300060520954763.
10. Ding, L., Y. Shen, A. Wang, C. Lu, X. Gu and L. Jiang (2022). "Construction of a novel miRNA regulatory network and identification of target genes in gestational diabetes mellitus by integrated analysis." Front Genet 13: 966296.
11. Feng, Y., X. Qu, Y. Chen, Q. Feng, Y. Zhang, J. Hu and X. Li (2020). "MicroRNA-33a-5p sponges to inhibit pancreatic β-cell function in gestational diabetes mellitus LncRNA DANCR." Reproductive Biology and Endocrinology 18(1): 1-9.
12. Filardi, T., G. Catanzaro, G. E. Grieco, E. Splendiani, S. Trocchianesi, C. Santangelo, R. Brunelli, E. Guarino, G. Sebastiani, F. Dotta, S. Morano and E. Ferretti (2022). "Identification and Validation of miR-222-3p and miR-409-3p as Plasma Biomarkers in Gestational Diabetes Mellitus Sharing Validated Target Genes Involved in Metabolic Homeostasis." Int J Mol Sci 23(8).
13. Ghaneialvar, H., M. M. Mohseni, A. Kenarkoohi and S. Kakaee (2024). "Are miR-26a and miR-26b microRNAs potent prognostic markers of gestational diabetes?" Health Sci Rep 7(6): e2152.
14. Gillet, V., A. Ouellet, Y. Stepanov, R. S. Rodosthenous, E. K. Croft, K. Brennan, N. Abdelouahab, A. Baccarelli and L. Takser (2019). "miRNA profiles in extracellular vesicles from serum early in pregnancies complicated by gestational diabetes mellitus." The Journal of Clinical Endocrinology & Metabolism 104(11): 5157-5169.
15. Gomez Ribot, D., E. Diaz, M. V. Fazio, H. L. Gómez, D. Fornes, S. B. Macchi, C. A. Gresta, E. Capobianco and A. Jawerbaum (2020). "An extra virgin olive oil-enriched diet improves maternal, placental, and cord blood parameters in GDM pregnancies." Diabetes/Metabolism Research and Reviews 36(8): e3349.
16. Guan, C.-Y., S. Tian, J.-L. Cao, X.-Q. Wang, X. Ma and H.-F. Xia (2020). "Down-regulated miR-21 in gestational diabetes mellitus placenta induces PPAR-α to inhibit cell proliferation and infiltration." Diabetes, Metabolic Syndrome and Obesity: Targets and Therapy 13: 3009.
17. Guan, C. Y., J. L. Cao, L. Zhang, X. Q. Wang, X. Ma and H. F. Xia (2022). "miR-199a Is Upregulated in GDM Targeting the MeCP2-Trpc3 Pathway." Front Endocrinol (Lausanne) 13: 917386.
18. He, H., Y. Wang, H. Wang, Y. Ma and P. Zhang (2024). "Correlation between serum microRNA-122 and VEGF expression and pregnancy outcome in gestational diabetes mellitus patients." Pak J Med Sci 40(3Part-II): 326-331.
19. He, Y., J. Bai, P. Liu, J. Dong, Y. Tang, J. Zhou, P. Han, J. Xing, Y. Chen and X. Yu (2017). "miR-494 protects pancreatic β-cell function by targeting PTEN in gestational diabetes mellitus." Excli j 16: 1297-1307.
20. Hromadnikova, I., K. Kotlabova and L. Krofta (2022). "Cardiovascular disease-associated microRNAs as novel biomarkers of first-trimester screening for gestational diabetes mellitus in the absence of other pregnancy-related complications." International Journal of Molecular Sciences 23(18): 10635.
21. Hu, Y., Q. Li, L. Zhang, L. Zhong, M. Gu, B. He, Q. Qu, Y. Lao, K. Gu, B. Zheng and H. Yang (2021). "Serum miR-195-5p Exhibits Clinical Significance in the Diagnosis of Essential Hypertension with Type 2 Diabetes Mellitus by Targeting DRD1." Clinics 76: e2502.

22. Hua, Z., D. Li, A. Wu, T. Cao and S. Luo (2021). "miR-377 inhibition enhances the survival of trophoblast cells via upregulation of FNDC5 in gestational diabetes mellitus." Open Medicine 16(1): 464-471.
23. Jamalpour, S., S. M. Zain, R. Vazifehmand, Z. Mohamed, Y. F. Pung, H. Kamyab and S. Z. Omar (2022). "Analysis of serum circulating MicroRNAs level in Malaysian patients with gestational diabetes mellitus." Sci Rep 12(1): 20295.
24. Ji, Y., W. Zhang, J. Yang and C. Li (2020). "MiR-193b inhibits autophagy and apoptosis by targeting IGFBP5 in high glucose-induced trophoblasts." Placenta 101: 185-193.
25. Jiang, Y., L. Wei, H. Zhang, Y. Chen, P. Gao, J. Zhang, X. Zhou, S. Zhu, Y. Du, C. Fang, J. Li, L. Feng, M. He, S. Wang and J. Yu (2022). "miR-17-5p Promotes Glucose Uptake of HTR8/SVneo Trophoblast Cells by Inhibiting TXNIP/NLRP3 Inflammasome Pathway." Diabetes Metab Syndr Obes 15: 3361-3374.
26. Joshi, A., R. Azuma, R. Akumuo, L. Goetzl and S. E. Pinney (2020). "Gestational diabetes and maternal obesity are associated with sex-specific changes in miRNA and target gene expression in the fetus." International Journal of Obesity 44(7): 1497-1507.
27. Juchnicka, I., M. Kuźmicki, M. Niemira, A. Bielska, I. Sidorkiewicz, M. Zbucka-Krętowska, A. J. Krętowski and J. Szamatowicz (2022). "miRNAs as Predictive Factors in Early Diagnosis of Gestational Diabetes Mellitus." Front Endocrinol (Lausanne) 13: 839344.
28. Kunysz, M., M. Cieśla and D. Darmochwał-Kolarz (2024). "Evaluation of miRNA Expression in Patients with Gestational Diabetes Mellitus: Investigating Diagnostic Potential and Clinical Implications." Diabetes Metab Syndr Obes 17: 881-891.
29. Lamadrid-Romero, M., K. Solís, M. Cruz-Reséndiz, J. Pérez, N. Díaz, H. Flores-Herrera, G. García-López, O. Perichart, E. Reyes-Muñoz and F. Arenas-Huertero (2018). "Central nervous system development-related microRNAs levels increase in the serum of gestational diabetic women during the first trimester of pregnancy." Neuroscience research 130: 8-22.
30. Légaré, C., V. Desgagné, K. Thibeault, F. White, A. A. Clément, C. Poirier, Z. C. Luo, M. S. Scott, P. Jacques, P. Perron, R. Guérin, M. F. Hivert and L. Bouchard (2022). "First Trimester Plasma MicroRNA Levels Predict Risk of Developing Gestational Diabetes Mellitus." Front Endocrinol (Lausanne) 13: 928508.
31. Légaré, C., V. Desgagné, K. Thibeault, F. White, A. A. Clément, C. Poirier, Z. C. Luo, M. S. Scott, P. Jacques, P. Perron, R. Guérin, M. F. Hivert and L. Bouchard (2024). "First-Trimester Plasmatic microRNAs Are Associated with Fasting Glucose Levels in Late Second Trimester of Pregnancy." Biomedicines 12(6).
32. Li, J., L. Song, L. Zhou, J. Wu, C. Sheng, H. Chen, Y. Liu, S. Gao and W. Huang (2015). "A microRNA signature in gestational diabetes mellitus associated with risk of macrosomia." Cellular Physiology and Biochemistry 37(1): 243-252.
33. Li, L., S. Wang, H. Li, J. Wan, Q. Zhou, Y. Zhou and C. Zhang (2018). "microRNA-96 protects pancreatic β-cell function by targeting PAK1 in gestational diabetes mellitus." Biofactors 44(6): 539-547.
34. Li, S. C., A. Monazzam, M. Razmara, X. Chu, P. Stålberg and B. Skogseid (2021). "MiR-486-3p was downregulated at microRNA profiling of adrenals of multiple endocrine neoplasia type 1 mice, and inhibited human adrenocortical carcinoma cell lines." Sci Rep 11(1): 14772.
35. Li, Y. and J. Zhuang (2021). "miR-345-3p serves a protective role during gestational diabetes mellitus by targeting BAK1." Experimental and therapeutic medicine 21(1): 1-1.
36. Liao, X., Z. Zhou and X. Zhang (2020). "Effects of miR-195-5p on cell proliferation and apoptosis in gestational diabetes mellitus via targeting EZH2." Molecular Medicine Reports 22(2): 803-809.
37. Lin, H., X. Chen, L. Wang, T. Zhu, X. Feng, X. Liu, H. Chen and S. Pan (2024). "Unraveling the role of microRNAs: potential biomarkers for gestational diabetes mellitus revealed through RNA sequencing analysis." Archives of Gynecology and Obstetrics: 1-10.
38. Liu, C., H. Feng, L. Zhang, Y. Guo, J. Ma and L. Yang (2023). "MicroRNA-143-3p levels are reduced in the peripheral blood of patients with gestational diabetes mellitus and influences pancreatic β-cell function and viability." Exp Ther Med 25(2): 81.

39. Liu, L., J. Zhang and Y. Liu (2021). "MicroRNA-1323 serves as a biomarker in gestational diabetes mellitus and aggravates high glucose-induced inhibition of trophoblast cell viability by suppressing TP53INP1." Experimental and Therapeutic Medicine 21(3): 1-1.
40. Marei, E. and H. Gabr Youssef (2020). "Evaluation of MicroRNA-16 and MicroRNA-221 in Serum and Placenta in Gestational Diabetes Mellitus: Correlation with Macrosomia." Egyptian Journal of Radiation Sciences and Applications 33(2): 107-118.
41. Muralimanoharan, S., A. Maloyan and L. Myatt (2016). "Mitochondrial function and glucose metabolism in the placenta with gestational diabetes mellitus: role of miR-143." Clin Sci (Lond) 130(11): 931-941.
42. Nair, S., D. Guanzon, N. Jayabalan, A. Lai, K. Scholz-Romero, P. Kalita de Croft, V. Ormazabal, C. Palma, E. Diaz, E. A. McCarthy, A. Shub, J. Miranda, E. Gratacós, F. Crispi, G. Duncombe, M. Lappas, H. D. McIntyre, G. Rice and C. Salomon (2021). "Extracellular vesicle-associated miRNAs are an adaptive response to gestational diabetes mellitus." J Transl Med 19(1): 360.
43. Nair, S., N. Jayabalan, D. Guanzon, C. Palma, K. Scholz-Romero, O. Elfeky, F. Zuñiga, V. Ormazabal, E. Diaz, G. E. Rice, G. Duncombe, T. Jansson, H. D. McIntyre, M. Lappas and C. Salomon (2018). "Human placental exosomes in gestational diabetes mellitus carry a specific set of miRNAs associated with skeletal muscle insulin sensitivity." Clin Sci (Lond) 132(22): 2451-2467.
44. Niu, S., K. Yu, W. Wang, X. Tan, H. Xin and M. Wu (2022). "The Expression and Clinical Value of miR-221 and miR-320 in the Plasma of Women with Gestational Diabetes Mellitus." Clin Lab 68(7).
45. Peng, H. Y., M. Q. Li and H. P. Li (2018). "High glucose suppresses the viability and proliferation of HTR-8/SVneo cells through regulation of the miR-137/PRKAA1/IL-6 axis." International journal of molecular medicine 42(2): 799-810.
46. Peng, H. Y., M. Q. Li and H. P. Li (2019). "MiR-137 Restricts the Viability and Migration of HTR-8/SVneo Cells by Downregulating FNDC5 in Gestational Diabetes Mellitus." Curr Mol Med 19(7): 494-505.
47. Pfeiffer, S., B. Sánchez-Lechuga, P. Donovan, L. Halang, J. H. M. Prehn, A. Campos-Caro, M. M. Byrne and C. López-Tinoco (2020). "Circulating miR-330-3p in Late Pregnancy is Associated with Pregnancy Outcomes Among Lean Women with GDM." Sci Rep 10(1): 908.
48. Pheiffer, C., S. Dias, P. Rheeder and S. Adam (2018). "Decreased expression of circulating miR-20a-5p in South African women with gestational diabetes mellitus." Molecular diagnosis & therapy 22: 345-352.
49. Qi, S. and X. Wang (2019). "Decreased Expression of miR-185 in Serum and Placenta of Patients with Gestational Diabetes Mellitus." Clin Lab 65(12).
50. Qiu, H., X. Liu, S. Yao, J. Zhou, X. Zhang and J. Du (2020). "Regulation and mechanism of miR-518d through the PPARα-mediated NF-κB pathway in the development of gestational diabetes mellitus." Journal of Diabetes Research 2020.
51. Qiu, M., Z. Shangguan and H. Zhang (2020). "Downregulation of miR-451a in plasma may promote prothrombin expression and contribute to high blood coagulation state in gestational diabetes mellitus." Tropical Journal of Pharmaceutical Research 19(10): 2079-2084.
52. Sebastiani, G., E. Guarino, G. E. Grieco, C. Formichi, C. Delli Poggi, E. Ceccarelli and F. Dotta (2017). "Circulating microRNA (miRNA) expression profiling in plasma of patients with gestational diabetes mellitus reveals upregulation of miRNA miR-330-3p." Frontiers in endocrinology 8: 345.
53. Serati, A., C. Novielli, G. M. Anelli, M. Mandalari, F. Parisi, I. Cetin, R. Paleari and C. Mandò (2023). "Characterization of Maternal Circulating MicroRNAs in Obese Pregnancies and Gestational Diabetes Mellitus." Antioxidants (Basel) 12(2).
54. Shah, K. B., S. D. Chernausek, A. M. Teague, D. E. Bard and J. B. Tryggestad (2021). "Maternal diabetes alters microRNA expression in fetal exosomes, human umbilical vein endothelial cells and placenta." Pediatric research 89(5): 1157-1163.
55. Song, F., A. Cai, Q. Ye, X. Chen, L. Lin and X. Hao (2021). "MiR-34b-3p impaired HUVECs viability and migration via targeting PDK1 in an in vitro model of gestational diabetes mellitus." Biochemical Genetics 59: 1381-1395.
56. Song, T. R., G. D. Su, Y. L. Chi, T. Wu, Y. Xu and C. C. Chen (2021). "Dysregulated miRNAs contribute to altered placental glucose metabolism in patients with gestational diabetes via targeting GLUT1 and HK2." Placenta 105: 14-22.

57. Sørensen, A. E., M. N. van Poppel, G. Desoye, P. Damm, D. Simmons, D. M. Jensen, L. T. Dalgaard and D. C. I. Group (2021). "The predictive value of miR-16,-29a and-134 for early identification of gestational diabetes: a nested analysis of the DALI cohort." Cells 10(1): 170.
58. Sørensen, A. E., M. N. M. van Poppel, G. Desoye, D. Simmons, P. Damm, D. M. Jensen, L. T. Dalgaard and G. The Dali Core Investigator (2022). "The Temporal Profile of Circulating miRNAs during Gestation in Overweight and Obese Women with or without Gestational Diabetes Mellitus." Biomedicines 10(2).
59. Stirm, L., P. Huypens, S. Sass, R. Batra, L. Fritsche, S. Brucker, H. Abele, A. M. Hennige, F. Theis and J. Beckers (2018). "Maternal whole blood cell miRNA-340 is elevated in gestational diabetes and inversely regulated by glucose and insulin." Scientific reports 8(1): 1-12.
60. Sun, D.-G., S. Tian, L. Zhang, Y. Hu, C.-Y. Guan, X. Ma and H.-F. Xia (2020). "The miRNA-29b Is Downregulated in Placenta During Gestational Diabetes Mellitus and May Alter Placenta Development by Regulating Trophoblast Migration and Invasion Through a HIF3A-Dependent Mechanism." Frontiers in Endocrinology 11.
61. Tagoma, A., K. Alnek, A. Kirss, R. Uibo and K. Haller-Kikkatalo (2018). "MicroRNA profiling of second trimester maternal plasma shows upregulation of miR-195-5p in patients with gestational diabetes." Gene 672: 137-142.
62. Toljic, M., N. Nikolic, I. Joksic, J. Carkic, J. Munjas, N. Karadzov Orlic and J. Milasin (2024). "Expression of miRNAs and proinflammatory cytokines in pregnant women with gestational diabetes mellitus." J Reprod Immunol 162: 104211.
63. Tryggestad, J. B., A. Vishwanath, S. Jiang, A. Mallappa, A. M. Teague, Y. Takahashi, D. M. Thompson and S. D. Chernausek (2016). "Influence of gestational diabetes mellitus on human umbilical vein endothelial cell miRNA." Clin Sci (Lond) 130(21): 1955-1967.
64. Tu, C., L. Wang, H. Tao, L. Gu, S. Zhu and X. Chen (2020). "Expression of miR-409-5p in gestational diabetes mellitus and its relationship with insulin resistance." Experimental and Therapeutic Medicine 20(4): 3324-3329.
65. Valerio, J., A. Barabash, N. Garcia de la Torre, P. De Miguel, V. Melero, L. Del Valle, I. Moraga, C. Familiar, A. Durán, M. J. Torrejón, A. Diaz, I. Jiménez, P. Matia, M. A. Rubio and A. L. Calle-Pascual (2022). "The Relationship between Serum Adipokines, miR-222-3p, miR-103a-3p and Glucose Regulation in Pregnancy and Two to Three Years Post-Delivery in Women with Gestational Diabetes Mellitus Adhering to Mediterranean Diet Recommendations." Nutrients 14(22).
66. Villota, S. D., M. Toledo-Rodriguez and L. Leach (2021). "Compromised barrier integrity of human feto-placental vessels from gestational diabetic pregnancies is related to downregulation of occludin expression." Diabetologia 64(1): 195-210.
67. Wander, P. L., E. J. Boyko, K. Hevner, V. J. Parikh, M. G. Tadesse, T. K. Sorensen, M. A. Williams and D. A. Enquobahrie (2017). "Circulating early-and mid-pregnancy microRNAs and risk of gestational diabetes." Diabetes research and clinical practice 132: 1-9.
68. Wang, F., Z. Li, M. Zhao, W. Ye, H. Wu, Q. Liao, S. Bu and Y. Zhang (2021). "Circulating miRNAs miR-574-5p and miR-3135b are potential metabolic regulators for serum lipids and blood glucose in gestational diabetes mellitus." Gynecological Endocrinology 37(7): 665-671.
69. Wang, F., W. Wang, L. Lu, Y. Xie, J. Yan, Y. Chen, C. Di, L. Gan, J. Si, H. Zhang and A. Mao (2020). "MicroRNA-16-5p regulates cell survival, cell cycle and apoptosis by targeting AKT3 in prostate cancer cells." Oncol Rep 44(3): 1282-1292.
70. Wang, J., Y. Pan, F. Dai, F. Wang, H. Qiu and X. Huang (2020). "Serum miR-195-5p is upregulated in gestational diabetes mellitus." Journal of Clinical Laboratory Analysis 34(8): e23325.
71. Wang, P., Z. Ma, Z. Wang, X. Wang, G. Zhao and Z. Wang (2021). "MiR-6869-5p induces M2 polarization by regulating PTPRO in gestational diabetes mellitus." Mediators of Inflammation 2021: 1-8.
72. Wang, P., H. Wang, C. Li, X. Zhang, X. Xiu, P. Teng and Z. Wang (2019). "Dysregulation of microRNA-657 influences inflammatory response via targeting interleukin-37 in gestational diabetes mellitus." Journal of Cellular Physiology 234(5): 7141-7148.
73. Wang, P., Z. Wang, G. Liu, C. Jin, Q. Zhang, S. Man and Z. Wang (2019). "miR-657 promotes macrophage polarization toward M1 by targeting FAM46C in gestational diabetes mellitus." Mediators of Inflammation 2019.

74. Wang, S., D. Wei, X. Sun, Y. Li, D. Li and B. Chen (2021). "MiR-190b impedes pancreatic β cell proliferation and insulin secretion by targeting NKX6-1 and may associate to gestational diabetes mellitus." Journal of Receptors and Signal Transduction 41(4): 349-356.
75. Wei, L., C. Cao, X. Ma, X. Wang, M. Wang and P. Zhang (2021). "Elevated Serum and Urine MiR-429 Contributes to the Progression of Gestational Diabetes Mellitus." Clin Lab 67(5).
76. Wen, J. and X. Bai (2021). "miR-520h Inhibits cell survival by targeting mTOR in gestational diabetes mellitus." Acta Biochimica Polonica 68(1): 65-70.
77. Xiao, Y., J. Ding, Y. Shi, L. Lin, W. Huang, D. Shen and W. Wang (2020). "MiR-330-3p contributes to INS-1 cell dysfunction by targeting glucokinase in gestational diabetes mellitus." J Obstet Gynaecol Res 46(6): 864-875.
78. Xu, K., D. Bian, L. Hao, F. Huang, M. Xu, J. Qin and Y. Liu (2017). "microRNA-503 contribute to pancreatic beta cell dysfunction by targeting the mTOR pathway in gestational diabetes mellitus." EXCLI journal 16: 1177.
79. Ye, Z., S. Wang, X. Huang, P. Chen, L. Deng, S. Li, S. Lin, Z. Wang and B. Liu (2022). "Plasma Exosomal miRNAs Associated With Metabolism as Early Predictor of Gestational Diabetes Mellitus." Diabetes 71(11): 2272-2283.
80. Yoffe, L., A. Polsky, A. Gilam, C. Raff, F. Mecacci, A. Ognibene, F. Crispi, E. Gratacós, H. Kanety and S. Mazaki-Tovi (2019). "Early diagnosis of gestational diabetes mellitus using circulating microRNAs." European journal of endocrinology 181(5): 565-577.
81. Yu, X., Z. Liu, J. Fang and H. Qi (2021). "miR-96-5p: A potential diagnostic marker for gestational diabetes mellitus." Medicine (Baltimore) 100(21): e25808.
82. Zhang, C., L. Wang, J. Chen, F. Song and Y. Guo (2020). "Differential expression of miR-136 in gestational diabetes mellitus mediates the high-glucose-induced trophoblast cell injury through targeting E2F1." International journal of genomics 2020.
83. Zhang, L., Q. Wu, S. Zhu, Y. Tang, Y. Chen, D. Chen and Z. Liang (2022). "Chemerin-Induced Down-Regulation of Placenta-Derived Exosomal miR-140-3p and miR-574-3p Promotes Umbilical Vein Endothelial Cells Proliferation, Migration, and Tube Formation in Gestational Diabetes Mellitus." Cells 11(21).
84. Zhang, L., T. Zhang, D. Sun, G. Cheng, H. Ren, H. Hong, L. Chen, X.-J. Jing, Y. Du and Y. Zou (2020). "Diagnostic Value of Dysregulated miRNAs in the Placenta and Circulating Exosomes for Gestational Diabetes Mellitus." Available at SSRN 3557974.
85. Zhang, Q., X. Ye, X. Xu and J. Yan (2023). "Placenta-derived exosomal miR-135a-5p promotes gestational diabetes mellitus pathogenesis by activating PI3K/AKT signalling pathway via SIRT1." Journal of Cellular and Molecular Medicine 27(23): 3729-3743.
86. Zhang, Y. and X. Chen (2020). "Dysregulation of microRNA-770-5p influences pancreatic-β-cell function by targeting TP53 regulated inhibitor of apoptosis 1 in gestational diabetes mellitus." Eur Rev Med Pharmacol Sci 24(2): 793-801.
87. Zhao, C., J. Dong, T. Jiang, Z. Shi, B. Yu, Y. Zhu, D. Chen, J. Xu, R. Huo and J. Dai (2011). "Early second-trimester serum miRNA profiling predicts gestational diabetes mellitus." PloS one 6(8): e23925.
88. Zhao, C., T. Zhang, Z. Shi, H. Ding and X. Ling (2014). "MicroRNA-518d regulates PPARα protein expression in the placentas of females with gestational diabetes mellitus." Molecular Medicine Reports 9(6): 2085-2090.
89. Zhao, C., C. Zhao and H. Zhao (2020). "Defective insulin receptor signaling in patients with gestational diabetes is related to dysregulated miR-140 which can be improved by naringenin." Int J Biochem Cell Biol 128: 105824.
90. Zhou, X., C. Xiang and X. Zheng (2019). "miR-132 serves as a diagnostic biomarker in gestational diabetes mellitus and its regulatory effect on trophoblast cell viability." Diagnostic pathology 14(1): 1-7.
